# Synergic interplay of the La motif, RRM1 and the interdomain linker of LARP6 in the recognition of collagen mRNA expands the RNA binding repertoire of the La module

**DOI:** 10.1093/nar/gku1287

**Published:** 2014-12-08

**Authors:** Luigi Martino, Simon Pennell, Geoff Kelly, Baptiste Busi, Paul Brown, R. Andrew Atkinson, Nicholas J.H. Salisbury, Zi-Hao Ooi, Kang-Wei See, Stephen J. Smerdon, Caterina Alfano, Tam T.T. Bui, Maria R. Conte

**Affiliations:** 1 Randall Division of Cell and Molecular Biophysics, King's College London, New Hunt's House, Guy's Campus, London SE1 1UL, UK; 2 Division of Molecular Structure, MRC National Institute for Medical Research, The Ridgeway, Mill Hill, London NW7 1AA, UK; 3 MRC Biomedical NMR Centre, MRC National Institute for Medical Research, The Ridgeway, Mill Hill, London NW7 1AA, UK; 4 Department of Biology, École Normale Supérieure de Lyon, CEDEX 07, France; 5 Department of Biological Sciences, National University of Singapore, Singapore 117543

## Abstract

The La-related proteins (LARPs) form a diverse group of RNA-binding proteins characterized by the possession of a composite RNA binding unit, the La module. The La module comprises two domains, the La motif (LaM) and the RRM1, which together recognize and bind to a wide array of RNA substrates. Structural information regarding the La module is at present restricted to the prototypic La protein, which acts as an RNA chaperone binding to 3′ UUU_OH_ sequences of nascent RNA polymerase III transcripts. In contrast, LARP6 is implicated in the regulation of collagen synthesis and interacts with a specific stem-loop within the 5′ UTR of the collagen mRNA. Here, we present the structure of the LaM and RRM1 of human LARP6 uncovering in both cases considerable structural variation in comparison to the equivalent domains in La and revealing an unprecedented fold for the RRM1. A mutagenic study guided by the structures revealed that RNA recognition requires synergy between the LaM and RRM1 as well as the participation of the interdomain linker, probably in realizing tandem domain configurations and dynamics required for substrate selectivity. Our study highlights a considerable complexity and plasticity in the architecture of the La module within LARPs.

## INTRODUCTION

**Figure 1.**
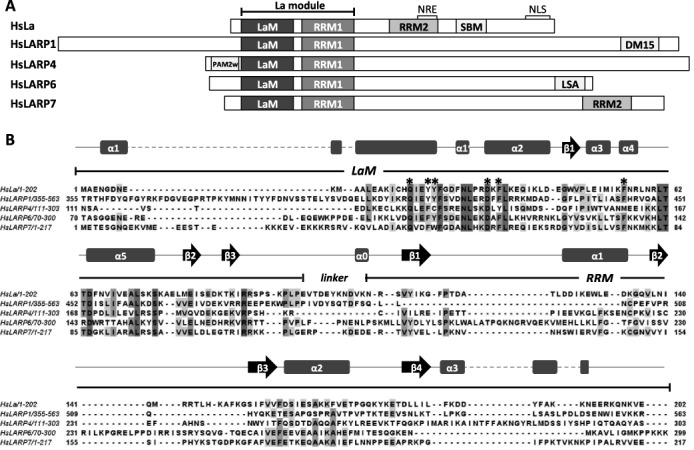
The LARPs. (A) Domain organization of human La and LARPs showing the conserved La module formed by the LaM and RRM1. Other domains/motifs are labelled as follows: RNA recognition motif 2 (RRM2); nuclear retention element (NRE); nuclear localization signal (NLS); short basic motif (SBM), DM15 box domain (DM15); variant PABP-interacting motif 2 (PAM2w); LaM and S1-like proteins associated motif (LSA) (1,2,11). The indicated domains/motifs are not in scale. (B) Multiple sequence alignment of the La modules of human LARPs performed with ClustalW2. The secondary structure elements of HsLa as well as the boundaries of HsLa LaM and RRM1 are indicated. The six highly conserved residues of the LaM, which in human La are involved in oligoU RNA binding, are labelled with asterisks.

The La-related proteins (LARPs) form a large and diverse superfamily of over 250 eukaryotic RNA-binding proteins that has emerged from recent phylogenetic analyses (1) and is divided into five distinct protein families: LARP1, La (aka LARP3), LARP4, LARP6 and LARP7 (Figure 1). Although LARPs perform a variety of discrete cellular functions (2), they share a conserved two-domain RNA binding unit, termed the ‘La module’, first identified in the La protein (3,4) and composed of a La motif (LaM), a novel type of winged-helix domain, and an RNA recognition motif (RRM1) (Figure 1). The La protein, which gives the name to this protein superfamily, has been studied for many years: it preferentially associates with RNA targets bearing a UUU stretch at the 3′ end, including all nascent RNA polymerase III (pol III) transcripts and a few short-lived pol II intermediate products, commensurate with its role as a chaperone in RNA biogenesis and metabolism for a number of precursor RNAs (2,5). The recognition of uridylate-containing 3′ ends by human La is achieved by a synergic interplay of the LaM and RRM1 whereby the two domains adopt an induced configuration around the RNA tailored for high-specificity binding to 3′ oligoU targets. The largest RNA interacting surface is comprised within a conserved hydrophobic pocket of the LaM, while RRM1's contacts with RNA are mainly confined to one edge of the strand β2 (4,6). Unexpectedly, neither the winged helix of the LaM nor the β-sheet surface of RRM1—the expected RNA binding surfaces of these domains—were seen to interact with 3′ oligoU RNA. Structural studies using a combination of nuclear magnetic resonance (NMR) and X-ray methodologies also revealed that RNA target discrimination in La is realized through the dynamic relationship of the two structurally independent domains within the La module connected by a flexible linker and this allows a significant degree of plasticity in the conformation of different bound RNA sequences (4).

An additional layer of complexity for this new RNA binding unit transpired when the interaction of human La with short oligoU sequences was found not to tell the full story: association of La with pre-tRNA transcripts, for example, involves contacts on the β-sheet RNA-binding surface (7) and, more intriguingly still, the binding of La to the internal ribosome entry site of Hepatitis C virus (HCV) RNA—which does not involve recognition of the RNA 3′ end—has revealed that the La module operates in conjunction with distal regions, in particular the RRM2 domain, to select RNAs of different sequence and shape (8).

Our appreciation of the significance of the La module was greatly enhanced when it was found in most LARPs where it provides an important central platform for RNA binding. The LaM in particular is highly conserved throughout the LARP superfamily and, although exceptions have been noted (2,9), primary sequence conservation extends to the six residues that in human La make specific contacts with 3′ UUU_OH_ (2,4). However, despite convergent features, unexpected RNA binding adaptability of the La module within the LARP superfamily has been emerging: whereas La binds specifically to 3′ UUU-OH single-stranded (ss) RNA, LARPs recognize different RNA targets, for example, human LARP6 binds to a specific stem-loop (SL) structure ((10) and this study), human LARP4 binds to ss polyA (11) and plant LARP6 proteins display different RNA binding properties from one another and from their eutherian counterpart (9). While consistent with the distinct functional roles of LARPs, the divergence in RNA substrate selection shows that structural models based on La are inadequate to understand LARP functions, and reaffirms that the non-canonical composite nature of the La module-RNA interactions makes it impossible at present to predict RNA binding based on protein sequence/structure alone. Notably, the La module of human La is to date the only available structure for this RNA binding unit.

In this study, therefore, we set out to investigate the molecular basis of the RNA binding properties of human LARP6. LARP6 (aka Acheron) is implicated in muscle differentiation and development in vertebrates (12,13). The mammalian protein is likely to have also a role in transcriptional regulation since it interacts with the developmental transcription factor CASK-C (14,15), although it remains uncertain whether these functions involve binding to RNA. However, in humans, where LARP6 is encoded by a single gene (9), RNA binding is unequivocally required for the regulation of the synthesis of collagen α1(I), α2(I) and α1(III) chains, which is mediated by a specific interaction between LARP6 and a SL structure in the 5′ untranslated region (5′ UTR) of these mRNAs (10,16). Disruption of this interaction decreases collagen production, making LARP6 an appealing target for treatment of fibroproliferative disorders (16,17).

We report here the determination of the solution structures of the LaM and RRM1 from human LARP6 (HsLARP6), revealing that both domains contain unusual features compared with human La. The RRM1 in particular was found to adopt an unprecedented fold for this domain class, bearing a novel α-helix in the loop β2/β3 obscuring the putative RNA binding site of the RRM. Mutagenesis analysis guided by the structures and informed by a recent phylogenetic study of the LARP6 family (9) was used, allied to biophysical techniques, to explore the RNA binding properties of human LARP6. The results show that mutations that disrupted the conserved hydrophobic crevice of the LaM or the LaM/RRM1 juxtaposition impaired the ability of LARP6 to interact with the SL of collagen mRNA. We suggest that the association of HsLARP6 with the SL sequence is mainly driven by non-electrostatic interactions and requires the synergic interplay of three components, the LaM, RRM1 and the connecting linker, programmed to act in a precise domain configuration to select their RNA substrates. This initial structural work on LARP6 sheds light on the mechanism of HsLARP6 interaction with collagen mRNA while paving the way for more incisive examination of the functional properties of LARP6 by establishing the boundaries and conformations of the RNA binding domains of the protein. Furthermore, this work provides a structural and conceptual framework for understanding the complexity of RNA recognition in proteins containing multiple RNA recognition moieties.

## MATERIALS AND METHODS

### Plasmid construction

Human LARP6 deletion mutants encompassing the LaM, HsLARP6(70–183), the RRM1, HsLARP6(180–295) and the La module, HsLARP6(70–300, 70–295 and 74–313), were amplified from full-length LARP6 using polymerase chain reaction (PCR) and subcloned into a pET-Duet1 vector (Novagen) with an N-terminal hexahistidine tag using standard methods as described elsewhere (18). Forward PCR primers used for producing his-tagged proteins encoded a TEV-cleavage site (ENLYFQG).

The alanine point substitution mutants of the La module of HsLARP6 (W85A, K86A, Q99A, F102A Y103A, D112A, F114A, F135A, L187A, Y189A, K196A, W198A, R231A, R237A, R244A, R245A, R249A, I260A, E262A), the single N180R and the triple R244E/R245E/R249E mutants were produced in the context of the HsLARP6(70–300) construct, using either the Quikchange approach (Stratagene) or the Overlap Extension PCR method (19). The latter was also used to generate the HsLARP6-HsLa chimera mutants, namely, Interlinker, Loop1, Loop3 and RRM1, which were designed as follows: in the Interlinker chimera mutant, the interdomain linker of HsLa (spanning residues 101–111) was introduced between residue 178 and 180 of HsLARP6; in the Loop1 chimera mutant, the loop 1 between strand β1 and helix α1 of the RRM1 of HsLARP6 (residues 190–208 based on Protein Data Bank (PDB) 2MTG reported in this manuscript) was replaced by the equivalent loop of HsLa RRM1 (residues 166–122 from PBD 1S79); in the Loop3 chimera mutant, the loop 3 between strands β2 and β3 of the HsLARP6 RRM1 (residues 234–257 based on PDB 2MTG reported in this manuscript) was replaced by the equivalent loop of HsLa RRM1 (residues 143–151 from PBD 1S79); in the RRM1 chimera mutant, the entire RRM1 of HsLARP6 (from residue 180) was exchanged with HsLa RRM1 (residues 111–202).

### Protein expression and purification

All the HsLARP6 proteins were expressed in *Escherichia coli* Rosetta II strain in rich media with induction by 1 mM IPTG (isopropyl β-D-thiogalactoside) at 18ºC for 14 h. For NMR, cells were grown on minimal media enriched with 0.8 g L^−1 15^N-ammonium chloride and 2 g L^−1 13^C glucose, and induced at 18ºC for 14 h. Cell pellets were resuspended in 50 mM Tris, pH 8.0, 300 mM NaCl, 10 mM imidazole, 5% glycerol, 2 mM PMSF (phenylmethanesulfonyl fluoride) and lysozyme, then lysed by sonication. Following centrifugation, the LARP6 proteins present in the soluble fraction were purified by affinity chromatography on a 5 mL HisTrap column (GE Healthcare) following the manufacturer's protocol. Proteins subjected to NMR, initial Isothermal titration calorimetry (ITC) tests and limited proteolysis underwent removal of the N-terminal His_6_-tag by overnight incubation with TEV^pro^ (at TEV^pro^:HsLARP6 molar ratio of 1:50) at 4°C in 50 mM Tris, pH 8.0, 100 mM KCl, 0.2 mM ethylenediaminetetraacetic acid (EDTA), 1 mM dithiothreitol (DTT). The reaction mixture was subsequently applied to a Ni-NTA column (Qiagen) to remove the cleaved tags, the His_6_-tagged TEV^pro^ and any undigested product, and the cleaved HsLARP6 protein was dialysed overnight in 50 mM Tris pH 7.25, 100 mM KCl, 0.2 mM EDTA, 1 mM DTT.

All the HsLARP6 proteins (with or without the His_6_-tag) were loaded onto a 5-mL Hi-Trap heparin column (GE Healthcare) mainly to eliminate nucleic acids contaminants, and eluted with a linear 0–2 M KCl gradient. The eluted proteins were dialysed in different buffers according to the subsequent experiment to be performed. Protein concentration was calculated based upon the near-ultraviolet (UV) absorption using theoretical extinction coefficients derived from ExPASY.

### RNA sample preparation

The 48 nt SL of the 5′ UTR of the collagen α1(I) mRNA and the modified 32 nt fragment were prepared by *in vitro* T7 polymerase transcription, using large-scale homogeneous RNA production performed as described (8). In brief, a 5′ hammerhead ribozyme and the target RNA sequences were cloned between the T7 promoter and the hepatitis δ ribozyme site in the plasmid pUC119δv, using XbaI and PstI restriction sites. The ribozyme constructs were linearized with *Hind*III and transcribed in a large-scale T7 polymerase reaction at 10–14 mL scale for 4 h at 37°C. The reaction mixtures were then annealed at 65°C for 10 min, slowly cooled to 55°C and held at this temperature for 30 min. The RNA samples were precipitated with sodium acetate and ethanol, then purified on 8 M urea 10% polyacrylamide denaturing gels and eluted with 0.5 M ammonium acetate, 10 mM magnesium acetate, 1 mM EDTA, 0.1% sodium dodecyl sulphate. Following ethanol precipitation, the RNA samples were extensively dialysed in water and lyophilized. The concentration of the dissolved oligonucleotides was evaluated by UV measurement at 95°C, using the molar extinction coefficients at 260 nm calculated by the nearest-neighbour model (20).

### Isothermal titration calorimetry

For most experiments protein and RNA solutions were prepared in 20 mM Tris, 100 mM KCl, 5 mM MgCl_2_, 1 mM DTT, pH 7.25 (exceptions are noted in the text and tables). Measurements were performed at 298 K using an ITC-200 microcalorimeter from Microcal (GE Healthcare) following the standard procedure reported previously (18). Typically, 20 injections of 2 μL of a solution containing 80–100 μM of HsLARP6 proteins were added into an RNA solution (8–10 μM) in the same buffer, using a computer-controlled 250-μL microsyringe. Integrated heat data obtained for the titrations corrected for heats of dilution were fitted using a non-linear least-squares minimization algorithm to a theoretical titration curve, using the MicroCal-Origin 7.0 software package. The fitting parameters were Δ*H*° (reaction enthalpy change in kcal·mol^−1^), *K*_b_ (equilibrium binding constant in M^−1^) and *n* (number of binding sites). The reaction entropy was calculated using the relationships Δ*G* = −*RT*·ln*K*_b_ (*R* 1.985 cal·mol^−1^·*K*^−1^, T 298 *K*) and Δ*G* = Δ*H*-*T*Δ*S*.

### Circular dichroism (CD)

CD spectra of RNA and protein samples were recorded on the Applied Photophysics Ltd. Chirascan Plus Spectrometer (Leatherhead, UK). Rectangular Suprasil cells with 1 cm path lengths were employed to record spectra in the regions between 340 and 220 nm. The parameters used to acquire the spectra were: spectral bandwidth of 1 nm, data step-size of 1 nm with a time-per-data-point of 1.5 s. Spectra were baseline corrected by subtracting the spectrum of the buffer alone. In all the experiments the protein concentration was in the range of 0.1–0.2 mg/ml (3.6–9 μM) and the RNA concentration was between 6 and 10 μM. The CD spectra of the protein-containing samples were acquired in 20 mM Tris, 100 mM KCl, 5 mM MgCl_2_, 1 mM DTT, pH 7.25.

### NMR spectroscopy

For NMR studies, HsLARP6 LaM, HsLARP6(70–183), and RRM1, HsLARP6(180–295), were concentrated to ∼0.5 mM in 20 mM Tris pH 7.25, 100 mM KCl, 50 mM arginine glutamate salt, 1 mM DTT and 20 mM Tris pH 7.25, 100 mM KCl, 1 mM DTT, respectively. The 50 mM L-Arg L-Glu was necessary to stabilize the HsLARP6 LaM protein solution at 298 K (21). NMR spectra were recorded at 298 K on a Varian Inova spectrometer operating 18.8 T and on Bruker Avance spectrometers at 14.1 and 16.4 T equipped with triple resonance cryoprobes. The ^1^H, ^15^N and ^13^C resonance assignments for HsLARP6 LaM and RRM1 will be reported elsewhere (*Biomol. NMR Assign.*, in preparation). All NMR data were processed using NMRPipe/NMRDraw (22) and analysed/assigned with CcpNMR analysis (23) and/or CARA/NEASY (24). Distance restraints used in structure calculation were obtained from ^1^H/^15^N- and ^1^H/^13^C-edited NOESY-HSQC experiments. Hydrogen-bonded amide protons were detected by recording a series of [^1^H,^15^N] HSQC experiments up to 10 h after the protein was buffer-exchanged in D_2_O. T1, T2 and [^1^H,^15^N] heteronuclear NOE relation experiments were recorded using pulse sequences adapted from standard schemes and analysed using NMRpipe. ^1^D_NH_ residual dipolar couplings for HsLARP6 RRM1 were measured at 298 K in a ternary complex composed of ∼4% (v/v) alkyl-poly(ethylene glycol) C8E5, ∼0.8% (v/v) *n*-octanol and 20 mM Tris-HCl, 100 mM KCl, 1 mM DTT pH 7.25. The liquid crystalline media gave a stable quadrupolar splitting of the D_2_O signal of about 30 Hz. The final concentration of the proteins in this media was about 0.18 mM. Precise measurements of ^1^J_NH_ splittings were obtained from in-phase/anti-phase (IPAP) [^1^H, ^15^N] HSQC experiments (25).

^1^H 1D NMR spectra were also recorded on HsLARP6(70–300), HsLARP6(70–295), HsLARP6(74–313), HsLARP6N180R and HsLARP6-HsLa Loop1 chimera in either 20 mM Tris pH 7.25, 100 mM KCl, 50 mM arginine glutamate salt, 1 mM DTT or 20 mM Tris pH 7.25, 100 mM KCl, 1 mM DTT.

[^1^H,^15^N] HSQC experiments of the La module, LARP6(70–300), were performed on a Bruker Avance spectrometer operating at 22.3 T and equipped with a triple resonance cryoprobe. The sample was concentrated to 80 μM in 20 mM Tris pH 7.25, 100 mM KCl, 50 mM arginine glutamate salt and 1 mM DTT and 20 mM Tris pH 7.25, 100 mM KCl, 1 mM DTT. The backbone amide resonances of the isolated LaM and RRM1 were transferred to the resonances of the La module that would have the smallest weighted chemical shift variation Δ*δ*_AV_, calculated as {0.5 [Δ*δ*(^1^H_N_)^2^ + (0.2 Δ*δ*(^15^N)]^2^}^1/2^.

### Structure calculation

Structure calculations of HsLARP6 LaM were performed with a restrained molecular dynamics-simulated annealing protocol executed in CNS (Crystallography and NMR System) 1.21 with ARIA (Ambiguous Restraints in Iterative Assignment) 2.3 (26). From ^15^N- and ^13^C-resolved 3D NOESY experiments, 1387 NOE distance restraints were obtained, including 569 intraresidue, 373 sequential (residue *i* to residue *i* + *j*, where *j* = 1), 221 medium-range (residue *i* to residue *i* + *j*, where 1 < *j* ≤ 4) and 223 long-range (residue *i* to residue *i* + *j*, where *j* > 4) NOEs. Sixty-nine key long-range NOEs were assigned manually and used to facilitate structure calculations. In addition, 174 ϕ/ψ dihedral angle restraints derived from TALOS+ (27) and 25 hydrogen bond distance restraints derived from proton exchange data were included in the calculation. Nine cycles of assignment/structure calculation were performed, using a standard protocol. The 20 lowest energy structures (over 100 calculated) from the last cycle represent the final family. Structure statistics are shown in Table 1.

**Table 1.** Structure calculation statistics for HsLARP6 LaM and RRM1

	LaM	RRM1
Residues	70–183	180–295
Number of models	20	20
Average rmsd (Å) among the 20 refined structures		
Backbone of structured regions^a^	0.69	0.56
Heavy atoms of structured regions^a^	1.19	1.06
Backbone of all residues^b^	0.99	0.80
Heavy atoms of all residues^b^	1.62	1.35
NMR restraints		
NOE restraints	1387	869
-Manual restraints	69	869
-Intraresidual (|i-j| = 0)	569	0
-Sequential (|i-j| = 1)	373	391
-Medium-range (1<|i-j| ≤ 4)	221	235
-Long-range (|i-j| > 4)	223	243
Hydrogen bonds	25	35
RDC	0	19
NOE restraints violations (>0.2 Å)	0.4 ± 0.2	none
NH residual dipolar coupling restraint violations > 2 Hz	-	none
Dihedral restraints	174	192
Dihedral restraint violations (> 5°)	none	none
Ramachandran statistics		
-Most favoured (%)	84.3%	82.8%
-Additionally allowed (%)	15.5%	14.1%
-Generously allowed (%)	0.2%	3.1%
-Disallowed (%)	0%	0%

^a^Structured regions selected on the basis of ^15^N backbone dynamics (Supplementary Figure S4). LaM: 85-118, 127-178; RRM1: 181-201, 210-291.

^b^LaM: residues 85-178; RRM1: residues 181-293.

For HsLARP6 RRM1 distance restraints were obtained from ^15^N- and ^13^C-edited 3D NOESY experiments and backbone dihedral angles were determined using TALOS+. The structures were calculated using a combined torsion angle and Cartesian coordinates dynamics protocol executed in CNS1.21 from random starting coordinates on the basis of 869 NOE distance restraints, including 391 sequential (residue *i* to residue *i* + *j*, where *j* = 1), 235 short-range (residue *i* to residue *i* + *j*, where 1 < *j* ≤ 4) and 243 long-range connectivities (residue *i* to residue *i* + *j*, where *j* > 4), 192 dihedral angle restraints (ϕ/ψ), 35 hydrogen-bond distance restraints and 19 residual dipolar coupling restraints. NOEs observed at 100 ms were classified as strong, medium or weak (<2.8, 3.8 and 5.5 Å, respectively) on the basis of peak intensities calibrated internally using known distances. The structures were analysed and displayed using PyMOL (www.pymol.org/) and MolMol (28). The final family, comprising the 20 structures of the lowest total energy from a total of 100 calculated structures, were inspected using Procheck *via* the Protein structure validation suite (http://psvs-1_4-dev.nesg.org/); structure statistics are shown in Table 1.

### Limited proteolysis

Proteins were subjected to limited tryptic proteolysis at a protein concentration of 0.5 μg/μL and a protein:trypsin ratio of 500:1 in 300 mM NaCl, 50 mM Tris pH 8 and 0.5 mM Tris(2-carboxyethyl)phosphine. Reactions were incubated at room temperature and 5 μg samples removed at intervals for analysis by gel electrophoresis. Where proteins were proteolyzed in the presence of RNA, 48 nt SL of the 5′ UTR of the collagen α1(I) mRNA was added at a 2-fold molar excess and the reactions pre-incubated at room temperature for 5 min prior to the addition of trypsin. Cleavage sites were identified by electrospray mass spectrometry of the proteolysis reaction mixtures.

### Protein sequence alignment

Protein sequence alignments were performed with ClustalW2 (http://www.ebi.ac.uk/Tools/msa/clustalw2/). The alignments were displayed and annotated using the Jalview software (29). Residues were shaded according to the extent of similarity.

## RESULTS

### Structure of the LaM and RRM1 of HsLARP6

**Figure 2.**
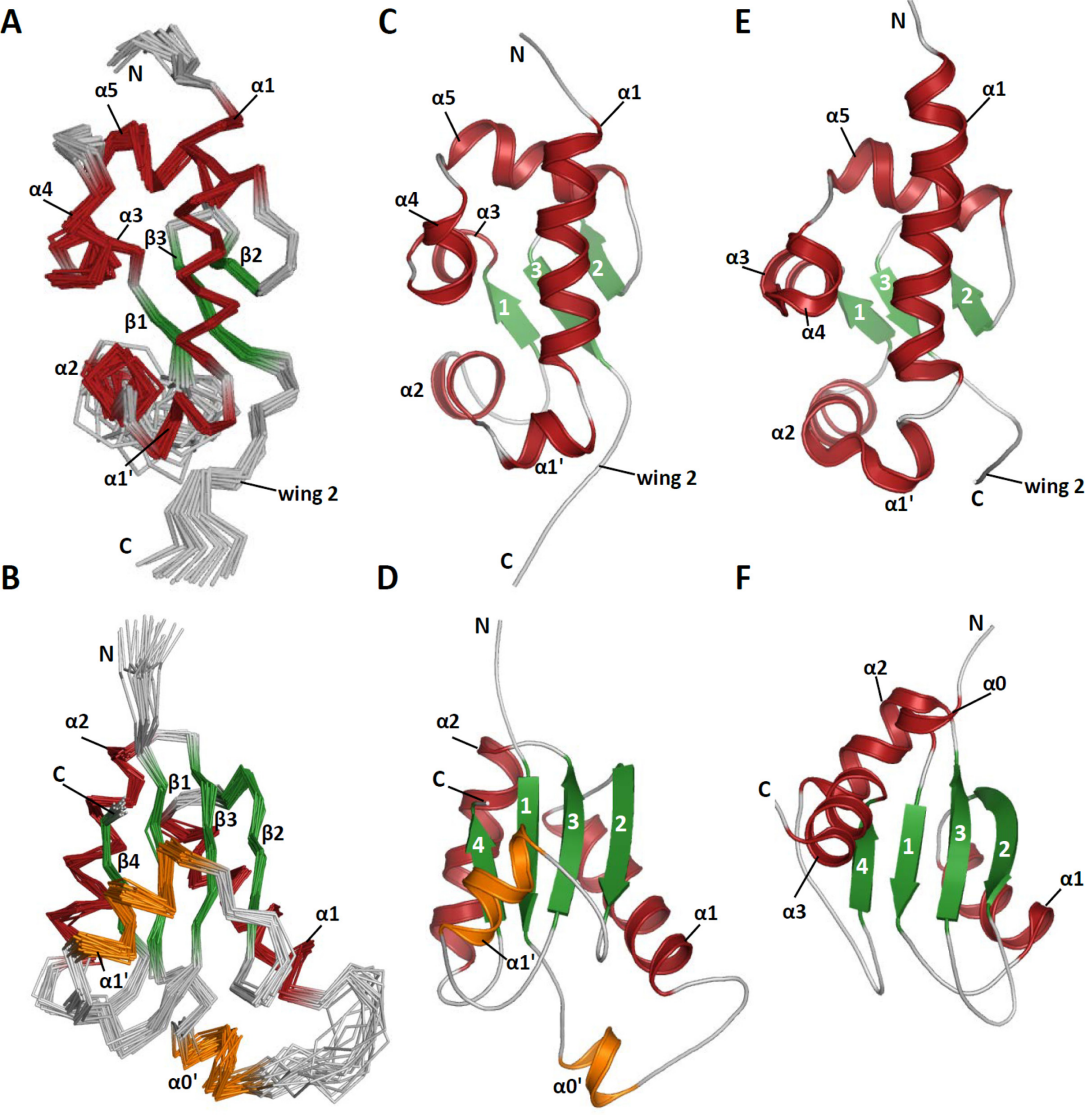
Structures of HsLARP6 LaM and RRM1. (A and B) Superposition of the backbone traces of the 20 lowest-energy structures for (A) the LaM and (B) the RRM1. The N- and C-termini, and the secondary structures are annotated. (C and D) Representative structures of (C) HsLARP6 LaM and (D) RRM1. (E and F) Structure of (E) HsLa LaM (PDB 1S7A) and (F) RRM1 (PDB 1S79) (3) displayed in the same orientation as HsLARP6 LaM and RRM1, respectively. α-helices are coloured in dark red and β-strands in green. β-strands are numbered in panels C–E. The novel α-helices in HsLARP6 RRM1 are reported in orange. All structure representations were generated using PyMOL.

Given that the minimal RNA binding domain in human LARP6 is the La module (see below), we first sought to determine the structure of this tandem domain. However, despite the numerous attempts, none of the available fragments spanning both the LaM and RRM1 produced a sample amenable to NMR or X-ray structure determination. We therefore chose to solve the structure of the isolated LaM and RRM1 domains, HsLARP6(70–183) and HsLARP6(180–295), respectively, where the superior sample stability and solubility allowed high resolution structure determination using standard heteronuclear multi-dimensional NMR techniques. For both domains, an ensemble of the 20 final structures with the lowest energy is reported in Figure 2 alongside a representative structure. Structure determination statistics are reported in Table 1.

#### HsLARP6 LaM

The overall structure of the LaM of human LARP6 closely resembles the homolog domain from the human La protein (Figure 2), which comes as no surprise given their high degree of sequence identity (33%). The DALI (30) output for HsLARP6 LaM structural homologs is topped by the LaM of La proteins from *Trypanosoma brucei* and *Homo sapiens* (*Z* score 9.1; rmsd 2.8 Å; identity 42%; PDB 1S29 and *Z* score 8.2, rmsd 3.5 Å; identity 33%; PDB 1S7A, respectively). As described previously, the LaM structure is an elaborated winged-helix domain, whereby three helical elements, namely, α1′, α2 and α4, are inserted onto the canonical fold (3,31). The RNA binding pocket of human La, containing the six residues that are well conserved across the species (Figure 1B and Supplementary Figure S1) and that in La are responsible for specific 3′ polyU recognition, assume a similar spatial arrangement in HsLARP6 LaM, with few exceptions (Supplementary Figure S2).

**Figure 3.**
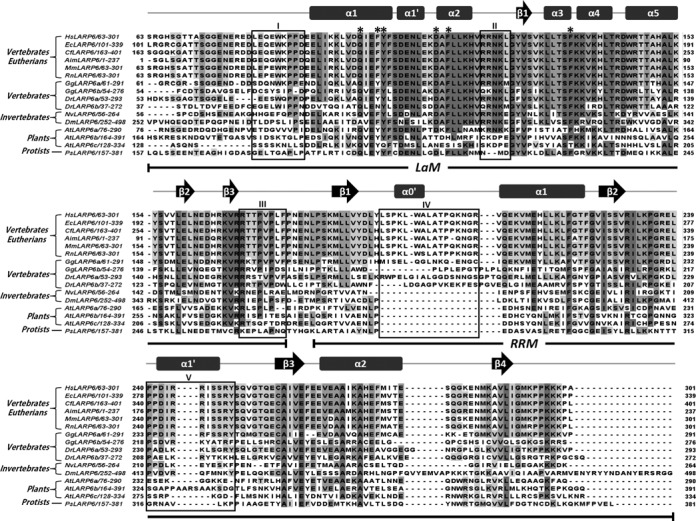
Alignment of LARP6 La modules from different species. The La module sequence of HsLARP6 was aligned with 15 LARP6 proteins from 11 species, including vertebrates-eutherians (*Equus caballus*, *Canis familiaris*, *Ailuropoda melanoleuca*, *Mus musculus*, *Rattus norvegicus*), vertebrates (*Gallus gallus*, *Danio rerio*), invertebrates (*Nematostella vectensis*, *Drosophila melanogaster*), plants (*Arabidopsis thaliana*) and protists (*Phytophtora sojae*). Secondary structure elements for HsLARP6 LaM/RRM1 and their domain boundaries (this study) are reported above and below the sequences, respectively. The six conserved residues on the LaM are indicated with asterisks. Boxes numbered from I to V indicate regions of structural/sequence dissimilarity between HsLARP6 and HsLa (see text). Species codes are the following: Ps, *Phytophtora sojae*; At, *Arabidopsis thaliana*; Nv, *Nematostella vectensis*; Dm, *Drosophila melanogaster*; Dr, *Danio rerio*; Gg, *Gallus gallus*; Aim, *Ailuropoda melanoleuca*; Cf, *Canis familiaris*; Mm, *Mus musculus*; Rn, *Rattus norvegicus*; Ec, *Equus caballus*; Hs, *Homo sapiens*.

Notably, the superposition of the HsLARP6 LaM structure with the corresponding domain of HsLa revealed some clear differences. First, helix α1 is shortened in HsLARP6 LaM, owing to the extended conformation adopted by residues 85–89, which lie across the upper surface of the protein flanking helix α5 and fixed in place by non-polar interactions of Trp85 with Arg146, Thr147 and His150 (Figure 2). While absent in other LARPs (Figure 1B), the stretch immediately preceding helix α1 of sequence ^81^LEQEWKPPD^89^ appears unique to the LaM of the LARP6 family where it is almost 100% conserved in the eutherian proteins, but diverging in invertebrates, plants and protists (Figure 3 and Supplementary Figure S3, box I).

The second element of dissimilarity between human LARP6 and La arises in the loop between α2 and β1, which in the former was found to be longer and less well defined. This region presumably undergoes conformational exchange at the milli-to-microsecond time scale because of the lack of backbone amide protons for residue 120–124 in the [^1^H,^15^N] HSQC spectra. This loop is well conserved across species aside from protists and a small subset of LARP6s from the green lineage (Figure 3 and Supplementary Figure S3, box II).

**Figure 4.**
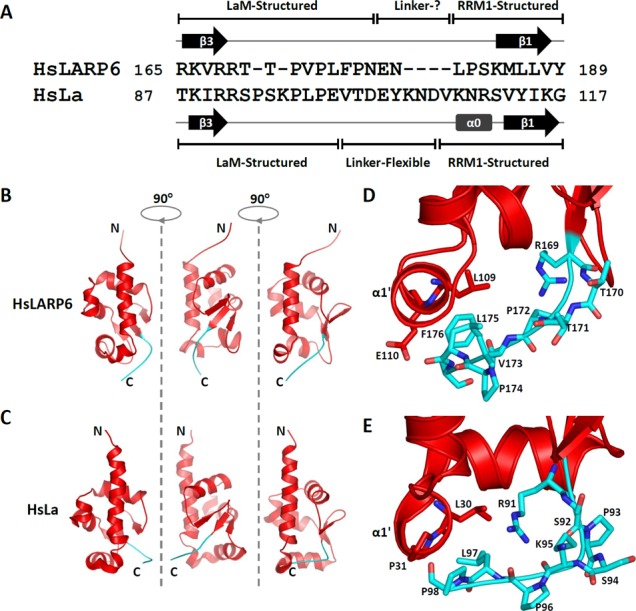
The wing 2 configuration is different in HsLARP6 and HsLa. (A) Alignment of selected amino acids encompassing the LaM wing 2 and interdomain linker for HsLARP6 and HsLa. The secondary structure elements as well as structured/flexible regions are indicated. For HsLa, this refers to the apo protein (4). (B and C) The conformation of the wing 2 highlighted in cyan is shown for HsLARP6 and HsLa LaM, respectively, in three different orientations. (D and E) Close-up view of the residues involved in forming and stabilizing the wing 2 relative to the rest of the LaM, for (D) HsLARP6 and (E) HsLa (see text).

Thirdly, and most interestingly, the configuration of the wing 2 differs in the LaM of HsLARP6 compared to HsLa, mirroring a divergence in the protein sequence for this region (Figure 4). Wing 2 is a structural facet of winged-helix proteins (32) demarcating a loop extending from strand β3 to the C-terminus of the domain, that in the majority of LaMs starts with two arginines and comprise the signature P(V/L)P motif (specifically ^90^RR^91^ and ^96^PLP^98^ in HsLa; ^168^RR^169^ and ^172^PVP^174^ in HsLARP6, respectively) (Figure 4A). In HsLa, the right angle bend of wing 2 positions the ^96^PLP^98^ tract of the LaM adjacent to helix α1′ thereby enabling hydrophobic contacts between the side chains of L97 and L30/P31 (Figure 4C and E). Structurally, the wing 2 of the LaM of La, and the domain itself, terminate with the ^96^PLP^98^ tract, with residues beyond P98 exhibiting flexibility and pointing away from the domain (3). Conversely, in human LARP6, although V173 (of the PVP tract) is likewise sited in the vicinity of the base of helix α1′, the well-structured C-terminal chain beyond ^172^PVP^174^ anchors to the LaM through alternative stabilising interactions involving L175/F176 of the wing 2 and L109/E110 of α1′ (Figure 4B and D). As a result, the domain boundaries of HsLARP6 LaM are shifted downstream, with residue 178 being the last structured residue of this domain (Supplementary Figure S4). The aminoacid sequence of the wing 2 aligns outstandingly well in LARP6 proteins from eutherians but conservation is progressively lost when moving away in evolutionary terms to the rest of the family (Figure 3 and Supplementary Figure S3, box III).

The surprising divergence of wing 2 configuration and LaM domain boundaries has a two-fold effect on LARP6 La module. First, given that residues up to 178 are integral part of the LaM structural core and that the RRM1 starts at residue 181 (see below), the interdomain linker is comprised by only two residues, E179 and N180 (Figure 4A), therefore shorter than it may appear from sequence alignment alone without knowledge of domain structure. Secondly, the end residue of the wing 2/LaM points in a different direction in HsLARP6 LaM compared to La (Figure 4B and C) and this may have repercussions on the reciprocal relationship between the LaM and RRM1 (see Discussion).

#### HsLARP6 RRM1

The HsLARP6 RRM1 (referred to as RRM-L3 in (1)) reveals an interesting new addition to the growing collection of RRM fold variations, whereby the canonical scaffold is elaborated by two new helices, termed α0′ and α1′ (Figure 2). The domain core is made up by the typical antiparallel four-stranded β-sheet flanked on one side by helices α1 and α2. Intriguingly, the opposite side of the β-sheet, housing the canonical RNA binding surface, is largely concealed by helix α1′, an unprecedented structural element comprised within the β2/β3 loop (aka loop 3). The packing of this helix against the domain is stabilized by a network of predominantly non-polar interactions engaging residues from the β-sheet including those belonging to the RRM hallmark sequences RNP-1 and RNP-2, specifically Ile243, Ile246 and Tyr250 from α1′ with Ile260 (RNP1), Leu187 and Tyr189 (RNP2) (Supplementary Figure S5).

Loop 3 has been frequently associated with RNA recognition in RRMs, but never to our knowledge found to adopt a helical conformation and to obscure the putative RNA binding surface on the β-sheet. Examples of α-helices performing this role have been described before, predominantly implicating N- and/or C-terminal helical extensions of the RRM fold, for instance, the La protein RRM2 (33) or Prp24 RRM4 (34). Of note, the solvent exposed face of helix α1′ in HsLARP6 RRM1 is decorated with basic residues (Supplementary Figure S6), and could therefore serve as a non-canonical RNA binding site (see below). The extent of primary structure conservation for α1′ rapidly decreases from the eutherian proteins to other species, calling into question whether this helix is present at all in plants, protists or even invertebrates (Figure 3 and Supplementary Figure S3, box V). Indeed, differences in loop 3 length were already appraised in the phylogenetic analysis of the LARP6 family (9).

An additional atypical trait of HsLARP6 RRM1 is the long loop between strand β1 and helix α1 (aka loop 1). A portion of this (residues 203–207) is ill defined in our structural model, largely reflecting a lack of chemical shift assignment for these residues, whereas amino acids 194–201 following on from strand β1 were found to fold into a short helix (termed α0′) which does not undergo internal motion according to our NMR relaxation analysis (Supplementary Figure S4). Once again, while extremely well conserved in eutherians, loop 1 is almost completely absent in protists, plants and inveterbrates. Interestingly, in vertebrates where LARP6 in encoded by 2 genes (termed a and b) the LARP6a subfamily appears to retain greater similarity to the eutherian proteins (Figure 3 and Supplementary Figure S3, box IV).

The DALI server identified the RRM of the yeast eukaryotic translation factor 3 as the closest structural neighbour of HsLARP6 RRM1, albeit the statistics do not endorse a high overall similarity (*Z* score 8.0; rmsd 2.8 Å; identity 15%; PDB 3NS5).

### Interaction of HsLARP6 with collagen mRNA 5′ UTR SL

**Figure 5.**
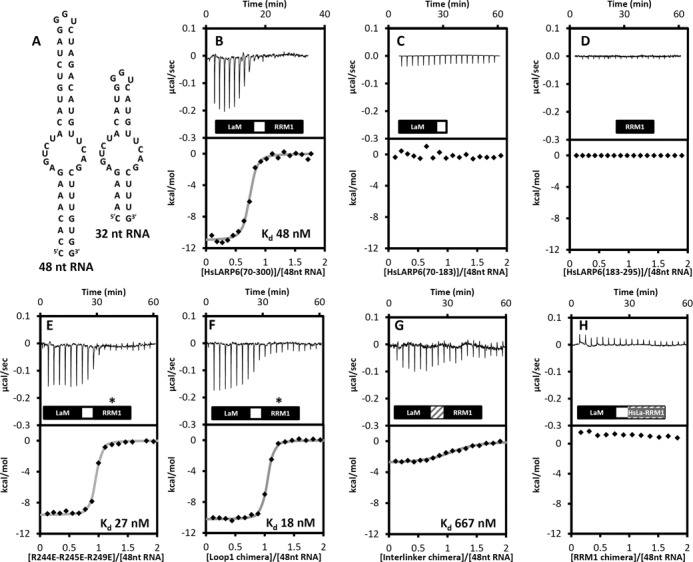
Interaction of HsLARP6 with RNA targets. (A) Expected secondary structures of the 48 nt and 32 nt SL RNAs obtained from mfold. (B–H) ITC experiments showing the thermal effect of mixing 48 nt RNA with (B) HsLARP6 La module (70–300), (C) HsLARP6 LaM, (D) HsLARP6 RRM1, (E) triple mutant R244E/R245E/R249E, (F) Loop1 chimera, (G) Interlinker chimera and (H) RRM1 chimera (see text). For each interaction the raw data and the normalized binding curve are reported. Black squares indicate the normalized heat of interaction obtained per each injection, while the grey curve represents the best fit obtained by a non-linear least-squares procedures based on an independent binding sites model. When measurable, dissociation constants are reported.

Human LARP6 was shown to bind to the 48 nt SL of the 5′ UTR of the collagen α1(I) mRNA (nucleotides 98–145, Figure 5A, hereafter referred to as 48 nt RNA) by the Stefanovic laboratory (10). In the same study the portion of the protein necessary and sufficient for this interaction was mapped to what we now know corresponds to an intact La module (residues 80–295) (10). To provide quantitative detail on the interaction of LARP6 with 48 nt RNA, we performed ITC measurements.

The La module of HsLARP6 was subjected to ITC titrations with 48 nt RNA, generating a well interpolated sigmoid-shaped curve based on an independent and equivalent binding sites model centred on a 1:1 stoichiometry and with a dissociation constant (*K*_D_) of 48 nM (Figure 5; Table 2). At 25°C, in the experimental conditions used the association is enthalpically driven with a small unfavourable entropic contribution (Table 2).

**Table 2.** Thermodynamic parameters of the association of human LARP6 and mutants thereof with 48, 32 and 4 nt oligoU RNA in 20 mM Tris pH 7.25, 100 mM KCl, 5 mM MgCl_2_, 1 mM DTT at 25°C

Interaction	*n*	*K*_d_ (nM)	Δ*H* (kcal)	-TΔ*S* (kcal)	Δ*G* (kcal)
HsLARP6(70-300)/48 nt RNA	0.8	48	−11	1.0	−10.0
HsLARP6(70-295)/48 nt RNA	1.1	77	−9.3	−0.4	−9.7
HsLARP6(70-183)/48 nt RNA	NB	NB	NB	NB	NB
HsLARP6(180-295)/48 nt RNA	NB	NB	NB	NB	NB
HsLARP6(74-313)/48 nt RNA	1.0	26	−9.7	−0.6	−10.3
HsLARP6(70-300)/32 nt RNA	1.0	125	−17	7.6	−9.4
HsLARP6(70-300)/UUUU_OH_ RNA	1.0	6000	−2.8	−4.1	−6.9

LaM mutations					
HsLARP6(70-300)W85A/48 nt RNA	0.9	22	−11	0.6	−10.4
HsLARP6(70-300)K86A/48 nt RNA	1.1	77	−11	1.3	−9.7
HsLARP6(70-300)Q99A/48 nt RNA	NB	NB	NB	NB	NB
HsLARP6(70-300)F102A/48 ntRNA	NB	NB	NB	NB	NB
HsLARP6(70-300)Y103A/48 nt RNA	NB	NB	NB	NB	NB
HsLARP6(70-300)D112A/48 nt RNA	0.9	172	−10	0.8	−9.2
HsLARP6(70-300)F114A/48 nt RNA	NB	NB	NB	NB	NB
HsLARP6(70-300)F135A/48 nt RNA	NB	NB	NB	NB	NB

Interdomain linker mutations					
HsLARP6(70-300)N180R/48 nt RNA	1.0	32	−13	2.8	−10.2
Interlinker chimera/48 nt RNA	1.1	667	−3.8	4.6	−8.4

RRM1 mutations					
HsLARP6(70-300)L187A/48 nt RNA	1.1	19	−11	0.5	−10.5
HsLARP6(70-300)Y189A/48 nt RNA	1.2	59	−13	3.1	−9.9
HsLARP6(70-300)K196A/48 nt RNA	1.0	48	−12	2.0	−10.0
HsLARP6(70-300)W198A/48 nt RNA	1.2	71	−13	3.3	−9.7
HsLARP6(70-300)R231A/48 nt RNA	1.1	20	−15	4.5	−10.5
HsLARP6(70-300)R237A/48 nt RNA	1.0	15	−14	3.3	−10.7
HsLARP6(70-300)R244A/48 nt RNA	1.1	71	−11	1.3	−9.7
HsLARP6(70-300)R245A/48 nt RNA	1.0	36	−9.5	−0.6	−10.1
HsLARP6(70-300)R249A/48 nt RNA	1.0	26	−8	−2.3	−10.3
HsLARP6(70-300)I260A/48 nt RNA	1.2	26	−13	2.7	−10.3
HsLARP6(70-300)E262A/48 nt RNA	0.9	5.3	−14	2.7	−11.3
Loop1 chimera/48 nt RNA	1.1	18	−11	−1.1	−9.9
R244E-R245E-R249E/48 nt RNA	1.1	27	−10	−0.3	−10.3
RRM1 chimera/48 nt RNA	NB	NB	NB	NB	NB

NB: no detectable binding.

The errors on the reported K_d_ and ΔH are between 5–15%.

For a quantitative comparison of the RNA selectivity within the LARP superfamily, HsLARP6(70–300) was also tested for its ability to interact with a 4 nt single stranded oligoU, the preferred RNA target of the La protein, revealing a ∼100-fold lower affinity for this ligand compared to the 48 nt RNA (Table 2). Consistent with a previous hypothesis implicating the predicted internal bulge of 48 nt SL in LARP6 binding (10), only a 2.6-fold difference in *K*_d_ was observed in the ITC measurements conducted with an RNA variant in which the length of the predicted double-stranded regions was shortened without affecting other structural elements (Figure 5, hereafter referred to 32 nt, Table 2). Notably, as no structural or biophysical information of the 48 and 32 nt SL is available to date, their conformation was scrutinized using a combination of CD and NMR spectroscopy as previously described (8) (data not shown) and found to be in agreement with the mfold predicted secondary structure (Figure 5).

A lesser role of the duplex RNA portion in LARP6 recognition is also in agreement with the lack of significant perturbations in the binding energetics of HsLARP6(70–300) for 48 nt SL RNA by varying concentrations of MgCl_2_ (0–10 mM) or KCl (100–200 mM) (Supplementary Table S1), in that the electrostatic contributions to binding, often associated with the duplex phosphate backbone recognition (8), appear negligible in this interaction.

### Correlating HsLARP6 structure to RNA recognition

We sought to identify residues/regions within the La module of human LARP6 responsible for its association with 48 nt RNA. An extensive panel of protein mutants was hence produced and assayed by ITC for their ability to bind the RNA ligand, with an emphasis on core conserved residues as well as atypical features revealed by our structural investigations. In each case, correct folding of the mutated proteins was verified by CD and/or NMR spectroscopy (data not shown).

All the mutants were generated in the context of HsLARP6 La module, residues 70–300. Akin to La (33), neither the LaM nor the RRM1 in isolation were found to bind 48 nt RNA, demonstrating that also in HsLARP6 both domains are strictly required for RNA recognition (Figure 5). Although a fragment of HsLARP6 encompassing residues 74–313 exhibited higher strand annealing chaperone activity compared to 70–300 (35), the additional C-terminal stretch beyond the RRM1 was found not to have an effect on the association with the 48 nt RNA (Table 2).

#### HsLARP6 LaM mutations

The 6 highly conserved residues that epitomize the LARP superfamily of proteins and line the hydrophobic crevice of both human La and LARP6 LaM (see above) were selected for site-directed mutagenesis to verify their role in LARP6-RNA recognition. Analogous to what was found for the La proteins from *T. brucei* (31) and human (6), 5 of these LARP6 mutations (Q99A, F102A, Y103A, F114A and F135A) significantly impaired RNA binding, whereas the D112A substitution had a significantly milder effect (Table 2, Supplementary Figure S7). These results suggest a key role of the LaM pocket in RNA binding for both La and LARP6 proteins, despite the very different RNA targets recognized in the two cases. Particularly puzzling is the case of D112: its counterpart in human La, D33, was found to be responsible for the specific 3′ end recognition of RNA by establishing bifurcated hydrogen bonds between its carboxylate side chain and the 2′ and 3′ OH of the terminal ribose (4,6). LARP6-RNA binding is 3′ OH independent, nevertheless D112 mutation to alanine yields a 3.5-fold reduction in the affinity for the 48 nt SL which bears a remarkable resemblance to the 2-fold affinity decrease observed for a HsLa D33A mutant with a 3′ oligoU ligand (6).

Next we turned our attention to the ^81^LEQEWKPPD^89^ motif highly conserved in eutherian LARP6 LaM (see above). Substitutions within this region (W85A, K86A), targeting residues commonly implicated in RNA recognition, did not perturb the interaction of LARP6 to the 48 nt RNA (Table 2, Supplementary Figure S7).

#### HsLARP6 RRM1 mutations

Contrary to the LaM, RRM1 domains are poorly conserved across the LARP superfamily as well as within the LARP6 proteins. The most common RNA recognition mechanism for RRMs entails stacking interactions between RNA bases and solvent exposed aromatic side chains in the conserved RNP-1 and RNP-2 sequences, located on strands β3 and β1, respectively (36). The predicted RNP stretches of human LARP6 RRM1, ^256^QECAIVEF^263^ and ^186^LLVYDL^192^, display, however, a poor match with consensus sequences and contain no aromatic residues at the 3 expected positions (36). Moreover, given that a number of side chains on the β-sheet engage in hydrophobic contacts with helix α1′ (see above), any involvement in RNA binding here would likely require a degree of displacement of α1′ from its original setting. The β-sheet surface of HsLARP6 RRM1 is also decorated with acidic amino acids (E256 and E262) that sometimes occur in other RRM domains albeit not at equivalent positions (37). Taking this into account, to sample the effect of β-sheet mutations on LARP6-RNA binding, alanine substitutions were performed for L187 (RNP-2), Y189 (RNP-2), I260 (RNP-1), E262 (RNP-1) and R231 (on β2), but none resulted in reduction of RNA binding activity. Interestingly, the E262A mutation enhanced the interaction with 48 nt SL 9-fold (Table 2, Supplementary Figure S7).

In addition to the β-sheet surface, loops interconnecting β-strands and α-helices can also play a part in RNA recognition by RRMs (36). We focused on loop 1 (β1/α1) and 3 (β2/β3), reasoning that their poor evolutionary conservation in the LARP6 family may at least in part account for the distinct RNA binding properties exhibited by different LARP6 proteins (9). Several single point mutants were engineered (R237A, R244A, R245A and R249A for loop 3; K196A and W198A for loop 1), on the grounds that basic residues in loop 3 have often been shown to form electrostatic interactions with the RNA sugar-phosphate backbone and that a role in RRM–RNA interaction has been reported for a single aromatic residue that is often present in the β1/α1 loop (loop 1) (36). None of these mutants, however, significantly altered the RNA binding profile of LARP6 for 48 nt RNA (Table 2). In view of the fact that single amino acid substitutions could potentially be compensated for in protein-RNA complexes exhibiting considerable molecular plasticity, we next replaced the entire loop 1 and 3 of human LARP6 with the respective counterparts of human La. The loop 1 swap did not perturb the interaction of LARP6 to the 48 nt SL (Table 2, Figure 5) while the effect of loop 3 substitution was not determined since the resultant recombinant protein was not expressed intact in *E. coli* cells. To probe further the role of the positive patch on helix α1′ in LARP6-RNA recognition, the triple R244E/R245E/R249E mutant was engineered, but once again the mutant retained the binding activity observed for HsLARP6(70–300) (Table 2, Figure 5). In a last effort to shed some light on the role of the RRM1 in LARP6-RNA recognition we produced a chimera mutant whereby the entire RRM1 of HsLARP6 was replaced by the RRM1 of HsLa. This drastic measure completely abolished RNA binding (Table 2, Figure 5), corroborating the importance of the specific LaM/RRM1 combination for RNA binding (see Discussion).

#### HsLARP6 interdomain linker mutations

Our experiments show that in HsLARP6 the tethered LaM and RRM1 domains work together as one RNA binding unit. We therefore hypothesized that the reciprocal domain distance, conformational dynamics and/or orientation would play a role in HsLARP6-RNA recognition and target discrimination, in line with what has previously been seen with the La protein and other modular RNA binding proteins exhibiting a cooperative behaviour (4,38).

The interdomain linker of HsLARP6 is a two-residue tract (see above). We followed a parallel strategy of generating a point mutant, N180R (designed also to be used in limited proteolysis analysis, see below), as well as exchanging the two residues with the 11 amino acid long linker from human La of sequence ^101^TDEYKNDVKNR^111^ (creating the ‘Interdomain linker chimera’). Whereas the single mutation did not impair binding, the binding efficiency of the chimera decreased 500-fold (Table 2, Figure 5). This marked effect may be attributable to an increased interdomain distance, a different spatial positioning and/or a change in conformational dynamics of the tandem domains (38), although a specific involvement of the linker in contacting the RNA cannot be excluded.

In an attempt to investigate this further, we turned to limited proteolysis studies of HsLARP6 La module in the presence and absence of 48 nt RNA, to probe for flexible or exposed regions in the apo/bound state, as well as to monitor any changes after conformational rearrangements elicited by ligand binding. A comparative analysis with the extensively studied human La protein served as benchmark to aid with the interpretation of experimental data. Whereas in HsLa the protease-sensitive interdomain linker becomes resistant to trypsin digest upon 3′ oligoU RNA interaction (Supplementary Figure S8), in accordance with previous NMR studies indicating a rigidification of this region following complex formation (4), the results for human LARP6 were inconclusive. As identified by mass spectrometry, trypsin cleaves HsLARP6 preferentially at the carboxyl side of K184 and R205 (Supplementary Figure S8), and this digestion pattern remained unchanged after RNA was added to the incubation mixture. Moreover, the introduction of a cleavage site for trypsin in the linker (i.e. N180R mutant) did not yield additional tryptic products, suggesting that the short HsLARP6 interdomain linker is not accessible to proteases.

Collectively, these results indicate that the interdomain linker plays a key role in RNA recognition of LARP6, although whether the loss of RNA binding affinity of the interdomain linker chimera is due to distance, orientation or dynamics of the composite domains remains unclear. The domain architecture of the La module in the apo form was further examined by NMR chemical shift analysis, whereby changes in the amide group resonances between the isolated LaM and RRM1 domains and the tandem La module could alert to the presence of stable interdomain interactions. Regrettably, the analysis was affected by poor La module sample stability, spectral overlap, line broadening, poor signal-to-noise ratio and concentration-dependent chemical shift variations. Nevertheless, whereas most of the backbone ^1^H and ^15^N resonances of the isolated domains were found not to vary in the La module, indicating that the structures of the two domains are largely retained in the tandem construct, we were able to identify a few signals that appear to experience chemical shift variation, suggesting that the LaM and RRM1 may not be fully independent from one another in the context of the free La module (Supplementary Figure S9). The perturbed residues thereby identified were mapped onto the structures of the isolated LaM and RRM1; however, they were not sufficient to delineate unambiguously potential surfaces of interaction between the two domains (data not shown). Further biophysical investigation to elucidate the structural and dynamic relationship of the LaM and RRM1 domains in the context of the tethered polypeptide (e.g. backbone relaxation analysis as for La (4)) were precluded by poor sample behaviour.

## DISCUSSION

In this paper we report a detailed investigation on the structure and RNA interactions of human LARP6. To date this is the first high resolution structure of the LaM and RRM1 of a La-related protein beyond the well-studied archetype La. On account of its high evolutionary conservation in the LARP superfamily, the LaM has traditionally been regarded as the invariant denominator for this family of proteins. The present investigations signal that this might be an oversimplified view, as variations in structure and domain boundaries across the LaMs, albeit small, do exist and could have a larger impact on RNA recognition than hitherto expected. By revealing different configurations for the N-terminal region preceding helix α1 and for the wing 2, our investigations of HsLARP6 LaM provide the exact boundaries for this domain that were not anticipated from sequence analyses. We showed that this LaM contains a structured insertion within its N-terminus (residues 85–89) and this promptly explains the loss of RNA binding activity observed in previous studies following inadvertent deletion of this portion (10), likely due to the unfolding of the domain. Even more intriguing is the unexpected variation uncovered for the wing 2 with its far-fetching repercussions on the juxtaposition of the LaM and RRM1 in the context of the La module (see below). The RRM1 on the other hand has generally been deemed as an unknown quantity in the LARP superfamily. Sequence alignment and structure prediction for this domain have been challenging, for example, the RRM1 domains in LARP6 were erroneously reported to lack the C-terminal β4 strand and the presence of RRM1 in a subset of LARPs is still debated (1). The high resolution structure determination of HsLARP6 RRM1 exposed an as yet unseen RRM-based fold, featuring an unprecedented version of loop 3, in that it contains an α-helix firmly seated on the canonical β-sheet face, probably precluding it from serving as the main RNA recognition platform of the domain. Interestingly, our structural data allied to phylogenetic analyses argue for a structural metamorphosis of the RRM1 in the LARP6 family through evolution, given that the novel structural features observed in the human protein (loops 1 and 3) do not correlate well with conservation at primary structure level. Although it would be plausible to hypothesize a link between evolutionary divergence and the different RNA binding properties seen in LARP6 proteins from different species ((9) and this study), the exact role of RRM structural diversity in LARP6-RNA recognition remains to be fully appreciated. All of the RRM1 mutants tested in this study, including those bearing major substitutions, were capable of recapitulating the binding behaviour of the wild-type HsLARP6 La module with the 48 nt SL RNA, and yet the replacement of the entire RRM1 with that of HsLa totally abrogated binding, endorsing the notion of specific—yet unidentified—features within HsLARP6 RRM1 dictating collagen mRNA recognition. It is noteworthy that, while the only one thus far identified, collagen 48 nt SL is unlikely to be the sole RNA target for LARP6, leaving the question open as to the non-conserved features in the RRM1 could play a role in the recognition of other substrates.

Interestingly, a couple of the RRM1 mutants investigated (*e.g.* E262A) displayed higher affinity for the 48 nt RNA compared to the wild-type HsLARP6 La module, albeit the significance of this observation is unclear at present.

Although it was anticipated that the mechanism of RNA recognition in LARP6 would entail synergism of the LaM and RRM1 by analogy with the La proteins (3,4,31), prior to our study this had not been conclusively demonstrated. Our mutagenesis analysis shows that RNA binding of HsLARP6 is compromised when the correct LaM/interdomain linker/RRM1 combination is altered, adding conviction to the view that each of these three components plays a distinctive role in La module RNA recognition. How might the interdomain linker contribute to RNA target selection in LARPs? In human La, the LaM, interdomain linker and RRM1 exist as an ensemble of quickly interconverting conformers in the apo state becoming ordered with respect to one another as RNA binding promotes protein compaction, with the linker correctly orienting the LaM and RRM1 to generate the RNA binding cleft (4). The short interdomain linker of LARP6 may also perform a topological role, spatially restricting local diffusion by defining a maximum distance between the LaM and RRM1 domains, which is significantly closer than in HsLa. In the absence of structural information for a tethered LaM and RRM1 within the HsLARP6 La module, we favour a model in which the short linker coupled with a different ‘exit’ of the LaM wing 2 would enforce a more elongated tandem domain arrangement and probably restrict the degree of conformational freedom of the LaM and RRM1 in the apo protein. In this scenario, a putative role of the linker might be to select for structural/dynamic alignments in which the two domains are ideally poised to recognize the cognate RNA (38). This hypothesis appears to be consistent with: (i) our preliminary chemical shift analysis suggesting interdomain interactions in the context of apo La module and (ii) the observation that replacement of the HsLARP6 linker with the longer one from HsLa, which would increase the domain–domain distance and perturb the degree of conformational sampling of the tandem domain in HsLARP6, has a detrimental effect on RNA binding.

**Figure 6.**
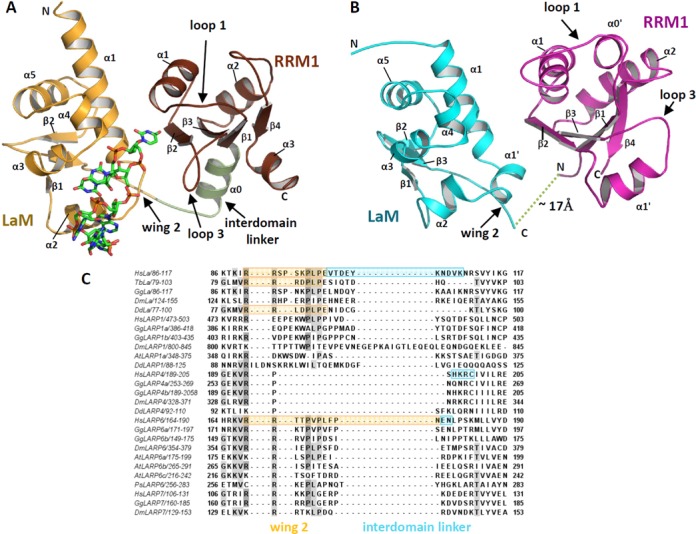
Sequence and structure divergence in the La modules from LARPs. (A) Structure of the La module of HsLa in complex with oligoU RNA (PDB 2VOD) (4). The LaM is coloured in yellow, the RRM1 in brown, the interdomain linker in green and the RNA is shown as sticks. (B) HsLARP6 LaM (in cyan - showing residues 85–178) and RRM1 (in magenta - residues 181–292) have been oriented with respect to one another analogously to the arrangement of the equivalent domains of HsLa in complex with oligoU (HsLARP6 LaM residues 90–119,125–169 were superposed to HsLa LaM residues 11–40,45–91; HsLARP6 RRM1 residues 185–189, 228–232, 258–263, 287–290 were superposed to HsLa RRM1 residues 112–116, 138–142, 153–158, 181–184). In this configuration, the distance from the last structured residue in the LaM to the first structured residue in the RRM1 in HsLARP6 is ∼17 Å, which will necessitate an inter-connecting linker in the order of 5–6 amino acids in a fully extended conformation. In absence of significant structural rearrangement of the individual domains upon RNA binding, the interdomain linker of HsLARP6 is only two residues long, making such tandem arrangement in HsLARP6 highly improbable. (C) Selected sequences encompassing the LaM wing 2 and the interdomain linker of HsLa were aligned with 25 LARP proteins from 6 different species including vertebrates-eutherians (*Homo sapiens*), vertebrates (*Gallus gallus*), invertebrates (*Drosophila melanogaster*), plants (*Arabidopsis thaliana*) and protists (*Dictyostelium discoideum*, *Phytophtora sojae*). Stretches experimentally demarcated as wing 2 and interdomain linker are indicated with a yellow and cyan box, respectively, revealing poor sequence alignment for these regions. Structural information was obtained from the following PDBs: HsLa (1S7A; 1S79; 2VON); TbLa (1S29); DdLa (2M5W); HsLARP6 (this study, 2MTF and 2MTG); HsLARP4 (MRC, unpublished results). Species codes are as for Figure 3 with the following addition: (Dd) *dictyostelium discoideum.*

Taken together, the data presented here indicate that, while the conserved hydrophobic pocket of the LaM in all probability serves as the main anchoring surface for the 48 nt RNA, RNA recognition by HsLARP6 requires the precise interlocking of the three-piece binding machinery that is the La module. Although a structural model of HsLARP6 in complex with RNA is yet to be worked out, our investigations suggest that the LaM and RRM1 in HsLARP6 will be unable to adopt the same side-by-side configuration as HsLa in a complex with RNA (Figure 6). A test of these ideas awaits the determination of the structure and dynamics behaviour of HsLARP6 La module in the free state and in complex with 48 nt RNA. Until that time, the definition of domain boundaries and the fold of the structured cores for HsLARP6 reported here provide an improved framework for the design of mutagenesis experiment to continue probing LARP6 functions.

Beyond LARP6, our findings can be used to reflect on other LARPs. A detailed inspection of the amino acid sequence prompted by our structural observations indicate that sequence divergences at the extremities of the LaM do exist across all the LARPs, most intriguingly involving the wing 2 region (Figure 6C, Supplementary Figure S1) with possible repercussions on RNA binding activity. Equally significant are the disparities we highlight here for the interdomain linker sequences/length across the LARP superfamily (Figure 6C), adding conviction to the view that this linker plays a key role in RNA target discrimination. Although a direct involvement of the linker region in targeting the RNA in LARPs cannot be ruled out, we propose that in many LARPs its main function would be to promote La module conformations competent for RNA binding, in some cases driving ligand association though reducing the entropic penalty for the association. The validation of these proposed mechanisms awaits further structural and biophysical investigations of other La modules.

Finally, it is noteworthy that the functional diversity of LARPs arises from the fact that the La modules are not only differentiated by their sequence but also by their placement within a distinct structural context (Figure 1A). This allows LARPs to interact with an array of diverse RNAs whereby ligand binding could also be mediated by other domains/motifs present in the protein working independently from the La module or in co-operation with it. The former situation is exemplified by the RRM2 of *Tetrahymena* p65 protein, which was shown to be sufficient for stem IV Telomerase RNA interaction (39), whereas the latter case is readily illustrated by the RRM2 of human La working in synergism with the La module to recognize structured RNAs, such as the SL IV of HCV IRES (8). Notably in LARP6, interaction with other RNA targets may involve the LSA (LaM and S1 Associated) motif, a nucleic acid-binding motif that was first observed appended to some cold-shock domains (1) (Figure 1A).

In conclusion, the present study strengthens and validates the concept of a three-piece modular construction for the La module across LARPs whereby the wildly diverse RNA binding properties observed in LARPs could be at least in part attributable to the correct LaM/linker/RRM1 combinatorial arrangement. This paves the way to a full understanding of the remarkable structural and RNA binding adaptability of the LARPs.

## ACCESSION NUMBERS

Coordinates and NMR structure calculation restraint files for HsLARP6 LaM and RRM1 have been deposited in the Protein Data Bank under accession codes 2MTF and 2MTG, respectively.

## SUPPLEMENTARY DATA

Supplementary Data are available at NAR Online.

SUPPLEMENTARY DATA
