# Supplementary material for: Synergic interplay of the La motif, RRM1 and the interdomain linker of LARP6 in the recognition of collagen mRNA expands the RNA binding repertoire of the La module
Source: Nucleic Acids Res. 2014 Dec 8;43(1):645–60. doi: 10.1093/nar/gku1287 (PMC4288179; doi:10.1093/nar/gku1287)
Supplement: SUPPLEMENTARY DATA [file supp_43_1_645__index.html]

Synergic interplay of the La motif, RRM1 and the interdomain linker of LARP6 in the recognition of collagen mRNA expands the RNA binding repertoire of the La module — SUPPLEMENTARY DATA 

# Synergic interplay of the La motif, RRM1 and the interdomain linker of LARP6 in the recognition of collagen mRNA expands the RNA binding repertoire of the La module

## SUPPLEMENTARY DATA

**Files in this Data Supplement:**

- SUPPLEMENTARY DATA
