# Supplementary material for: Synergic interplay of the La motif, RRM1 and the interdomain linker of LARP6 in the recognition of collagen mRNA expands the RNA binding repertoire of the La module
Source: Nucleic Acids Res. 2014 Dec 8;43(1):645–60. doi: 10.1093/nar/gku1287 (PMC4288179; doi:10.1093/nar/gku1287)
Supplement: SUPPLEMENTARY DATA [file supp_gku1287_nar-03195-z-2014-File003.pdf]

Supplementary Material

Synergic interplay of the La motif, RRM1, and the interdomain linker of LARP6 in the recognition of collagen mRNA expands the RNA binding repertoire of the La module

Luigi Martino[1], Simon Pennell[2], Geoff Kelly[3], Baptiste Busi[1,4], Paul Brown[1], R. Andrew Atkinson[1], Nicholas J.H. Salisbury[1], Zi-Hao Ooi[1,5], Kang-Wai See[1,5], Stephen J. Smerdon[2], Caterina Alfano[1], Tam T.T. Bui[1], Maria R. Conte[1]*

[1]Randall Division of Cell and Molecular Biophysics, King's College London, New Hunt's House, Guy's Campus, London SE1 1UL, UK

[2]Division of Molecular Structure and [3]MRC Biomedical NMR Centre, MRC National Institute for Medical Research, The Ridgeway, Mill Hill, London NW7 1AA, UK

[4]Department of Biology, École Normale Supérieure de Lyon, CEDEX 07, France

[5]Department of Biological Sciences, National University of Singapore, Singapore 117543

*To whom correspondence should be addressed. Tel. +44 207 8486194; Fax: +44 207 8486435. Email: sasi.conte@kcl.ac.uk

Figure S1. Multiple sequence alignment of LARP proteins from different species. The sequence encompassing the La module of HsLa was aligned with 22 LARP proteins from 6 different species including vertebrates-eutherians (*homo sapiens*), vertebrates (*Gallus gallus*), invertebrates (*Drosophila melanogaster*), plants (*Arabidopsis thaliana*) and protists (*Dictyostelium discoideum, Phytophtora sojae*). The alignment was performed with ClustalW2 (http://www.ebi.ac.uk/Tools/msa/clustalw2/). The boundaries of the LaM and the RRM1 for HsLa are indicated. Species codes are the following: (Ps) *Phytophtora species*; (Dd) *dictyostelium discoideum*; (At) *Arabidopsis thaliana*; (Dm) *Drosophila melanogaster*; (Gg) *Gallus gallus*; (Hs) *Homo sapiens*.

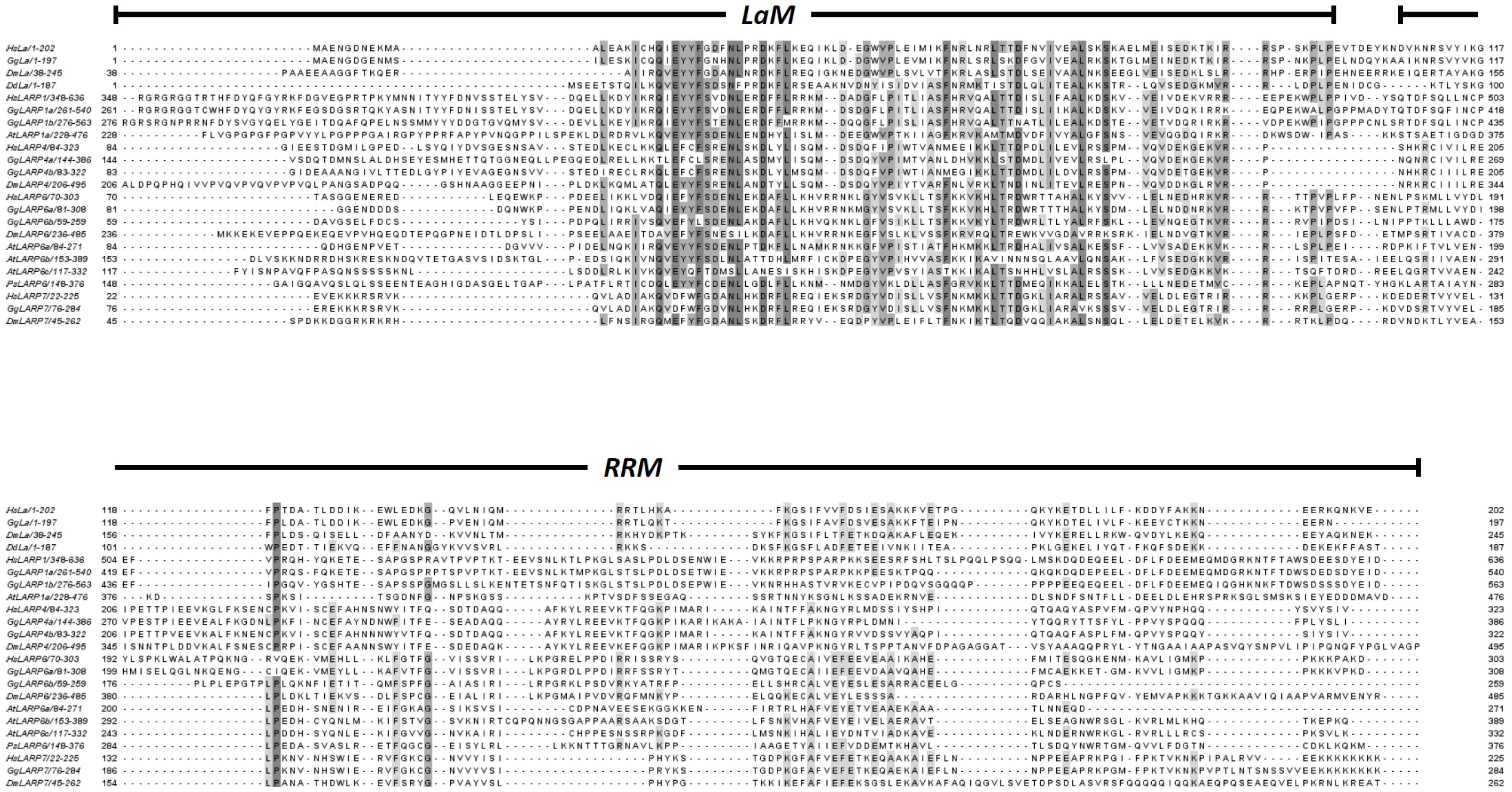
LaM
RRM
HsLa/1-202
GgLa/1-197
DmLa/38-245
DdLa/1-187
HsLARP1/348-636
GgLARP1a/261-540
GgLARP1b/276-563
AtLARP1a/228-476
HsLARP4/84-323
GgLARP4a/144-386
GgLARP4b/83-322
DmLARP4/206-495
HsLARP6/70-303
GgLARP6a/81-308
GgLARP6b/59-259
DmLARP6/236-485
AtLARP6a/84-271
AtLARP6b/153-389
AtLARP6c/117-332
PsLARP6/148-376
HsLARP7/22-225
GgLARP7/76-284
DmLARP7/45-262

Figure S2

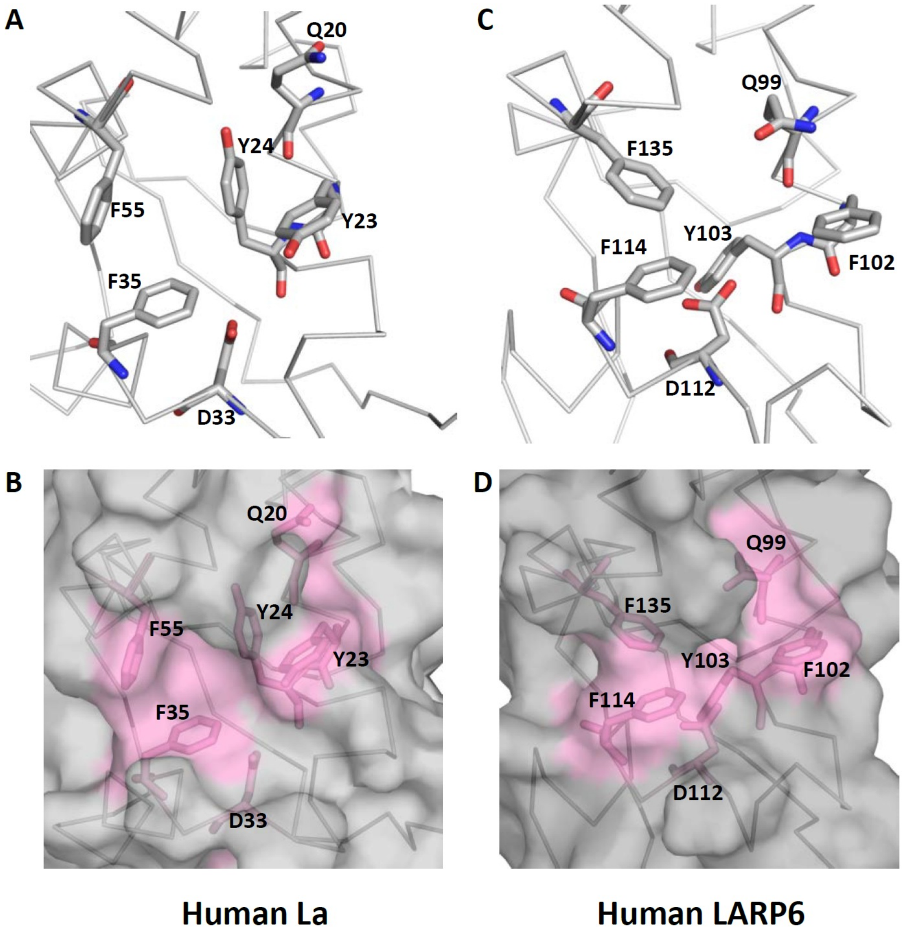

Figure S2. Analysis of the hydrophobic pocket of the LaM. Comparison of the hydrophobic crevice of the LaM in the apo state of (A,B) HsLa (PDB ID 1S7A) and (C,D) HsLARP6 (this study, PDB 2MTF). The residues that are extremely well conserved across LARPs and are important for RNA interaction are labelled and shown as sticks. The solvent exposed surface of the six conserved residues is coloured in pink in B and D. The side chain moieties of Q99, F102, D112 and F114 of HsLARP6 recapitulate the orientation found for the analogous residues in HsLa (Q20, Y23, D33, F35) while a different side chain orientation is observed for Y103 and F135 compared to HsLa Y24 and F55.

Figure S3

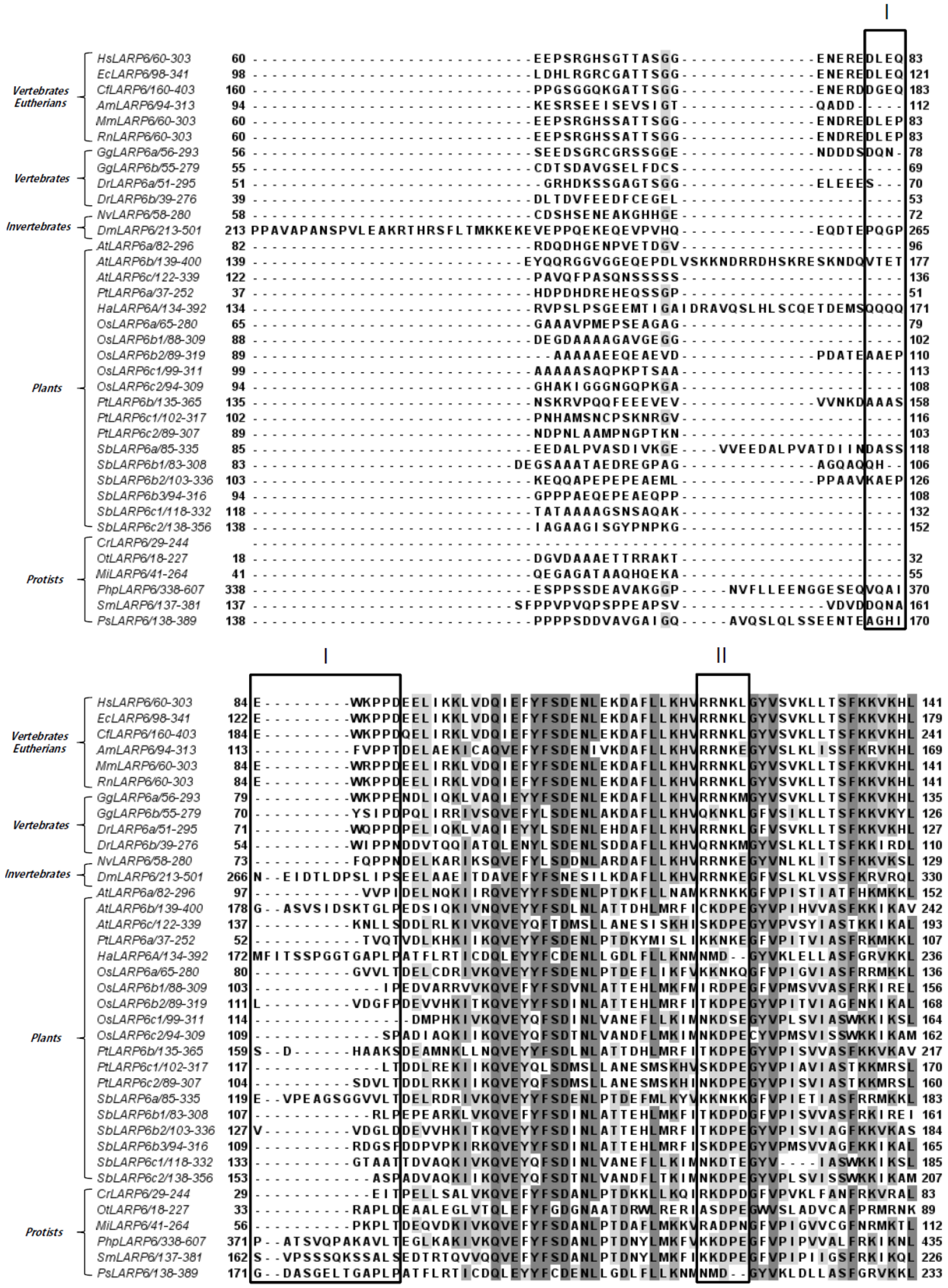
Vertebrates Eutherians
Vertebrates
Invertebrates
Plants
Protists
I
II
HsLARP6/60-303
EcLARP6/98-341
CfLARP6/160-403
AmLARP6/94-313
MmLARP6/60-303
RnLARP6/60-303
GgLARP6a/56-293
GgLARP6b/55-279
DrLARP6a/51-295
DrLARP6b/39-276
NvLARP6/58-280
DmLARP6/213-501
AtLARP6a/82-296
AtLARP6b/139-400
AtLARP6c/122-339
PtLARP6a/37-252
HaLARP6A/134-392
OsLARP6a/65-280
OsLARP6b1/88-309
OsLARP6b2/89-319
OsLARP6c1/99-311
OsLARP6c2/94-309
PtLARP6b/135-365
PtLARP6c1/102-317
PtLARP6c2/89-307
SbLARP6a/85-335
SbLARP6b1/83-308
SbLARP6b2/103-336
SbLARP6b3/94-316
SbLARP6c1/118-332
SbLARP6c2/138-356
CrLARP6/29-244
OtLARP6/18-227
MiLARP6/41-264
PhpLARP6/338-607
SmLARP6/137-381
PsLARP6/138-389

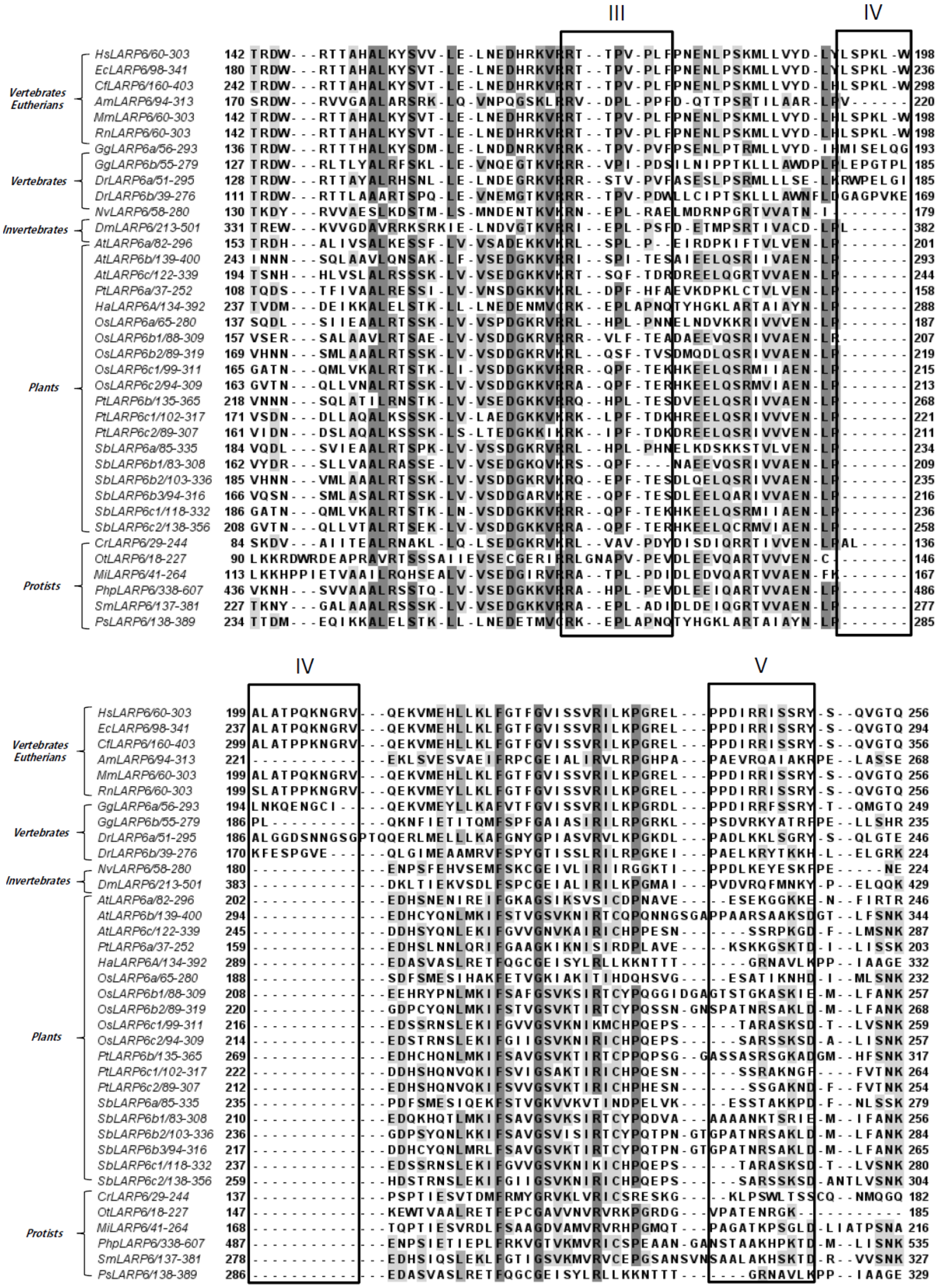

Figure S3: Alignment of LARP6 La modules from different species. The La module sequence of HsLARP6 was aligned with 36 LARP6 proteins from 21 species, including vertebrates-eutherians (*Equus caballus, Canis familiaris, Ailuropoda melanoleuca, Mus musculus, Rattus norvegicus*), vertebrates (*Gallus gallus, Danio*

rerio), invertebrates (*Nematostella vectensis, Drosophila melanogaster*), plant (*Arabidopsis thaliana, Populus trichocarpa, Hyaloperonospora arabidopsidis, Oryza sativa, Popolus trichocarpa, Sorghum bicolor*) and protist (*Chlamydomonas reinhardii, Osteococcus tauri, Micromonas, Physcomitrella patens, Selaginella maellendorfii, Phytophtora sojae*). Boxes numbered from I to V indicate regions of structural/sequence dissimilarity between HsLARP6 and HsLa (see text). Species codes are the following: (*Eq) Equus caballus, (Cf) Canis familiaris, (Am) Ailuropoda melanoleuca, (Mm) Mus musculus, (Rn) Rattus norvegicus*, (Gg) *Gallus gallus, (Dr) Danio rerio*, (Nv) *Nematostella vectensis, (Dn) Drosophila melanogaster*, (At) *Arabidopsis thaliana, (Pt) Populus trichocarpa, (Ha) Hyaloperonospora arabidopsidis, (Os) Oryza sativa, (Pt) Popolus trichocarpa, (Sb) Sorghum bicolor,* (Cr) *Chlamydomonas reinhardii, (Ot) Osteococcus tauri, (Mi) Micromonas, (Php) Physcomitrella patens, (Sm) Selaginella maellendorfii, (Ps) Phytophtora sojae*.

Figure S4

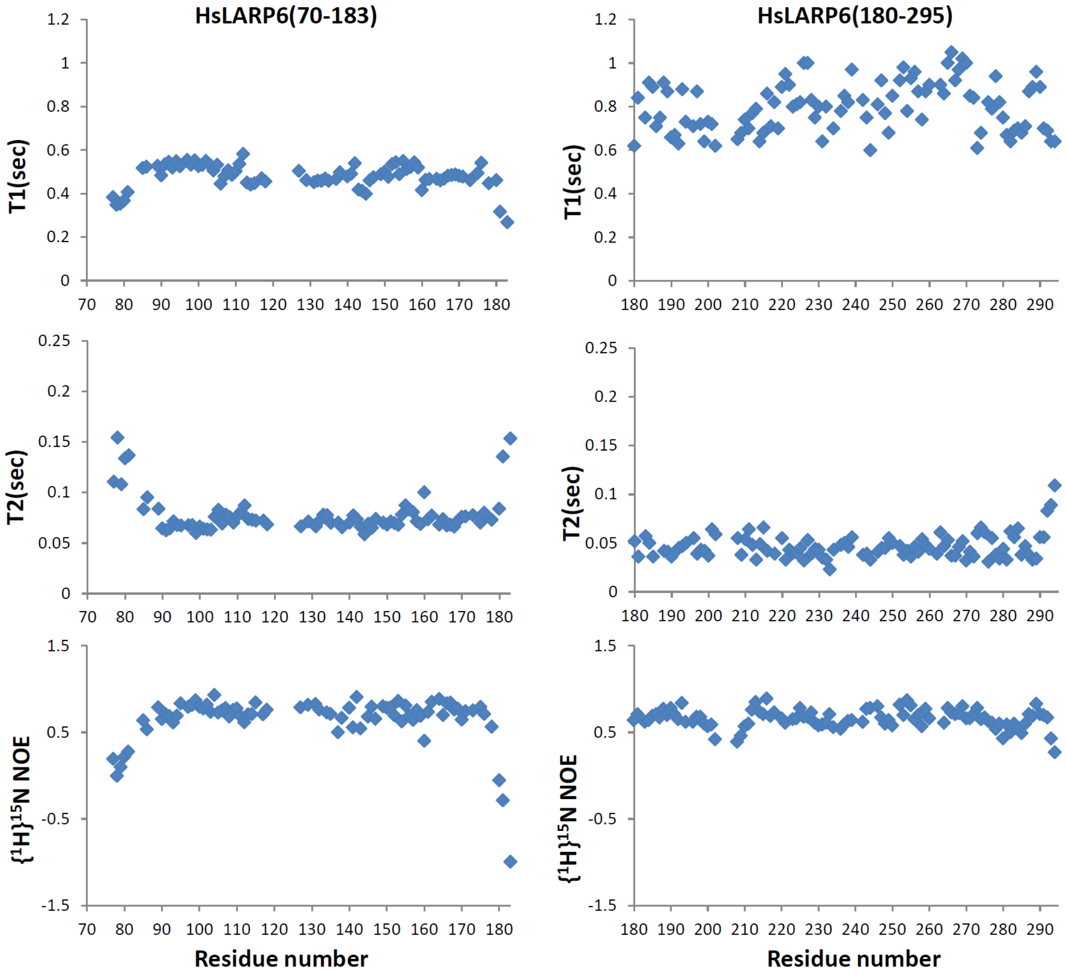

Figure S4. ^{15}N backbone dynamics of HsLARP6. ^{15}N relaxation analysis showing T1, T2 and {^{1}H}^{15}N NOE values for HsLARP6 LaM (70-183) and RRM1 (180-295) under the experimental conditions described in the Methods. The experiments were performed at 500 MHz for the LaM and 700 MHz for the RRM1. The gap in the LaM and RRM1 data partially reflects the lack of assignment for residues 120-124 and 203-207 respectively. The structured regions were found to be as follows: LaM: 85-118, 127-178; RRM1: 181-201, 210-291.

Figure S5

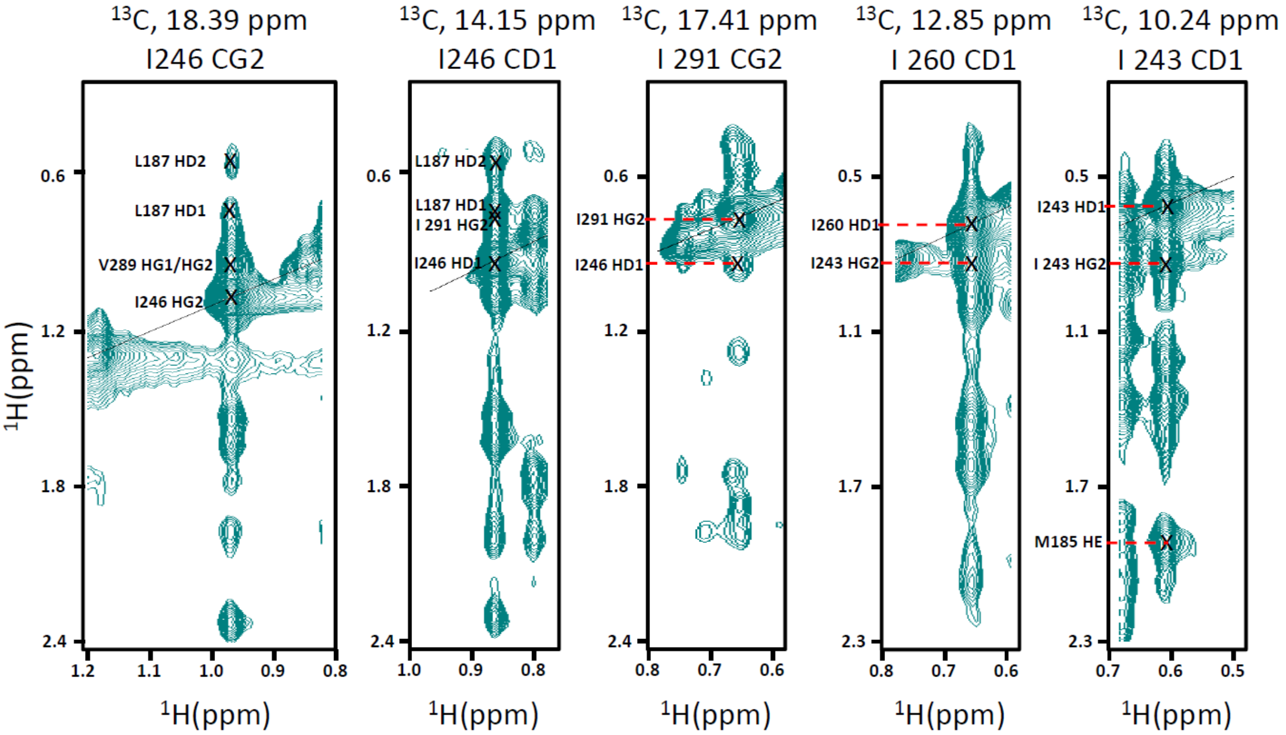

Figure S5: **Unambiguous NOEs involving residues of HsLARP6 RRM1 loop 3**. Representative planes of a ^{13}C-NOESY experiment recorded on HsLARP6(180-295) sample at 298 K on a Varian Inova spectrometer operating 18.8 T with a mixing time of 120 ms showing some of the unambiguous NOEs between protons belonging to the residues of the loop 3 α1’ (I246 and I243) and those onto the β-sheet (L187, V289, I291, I260, M185).

Figure S6

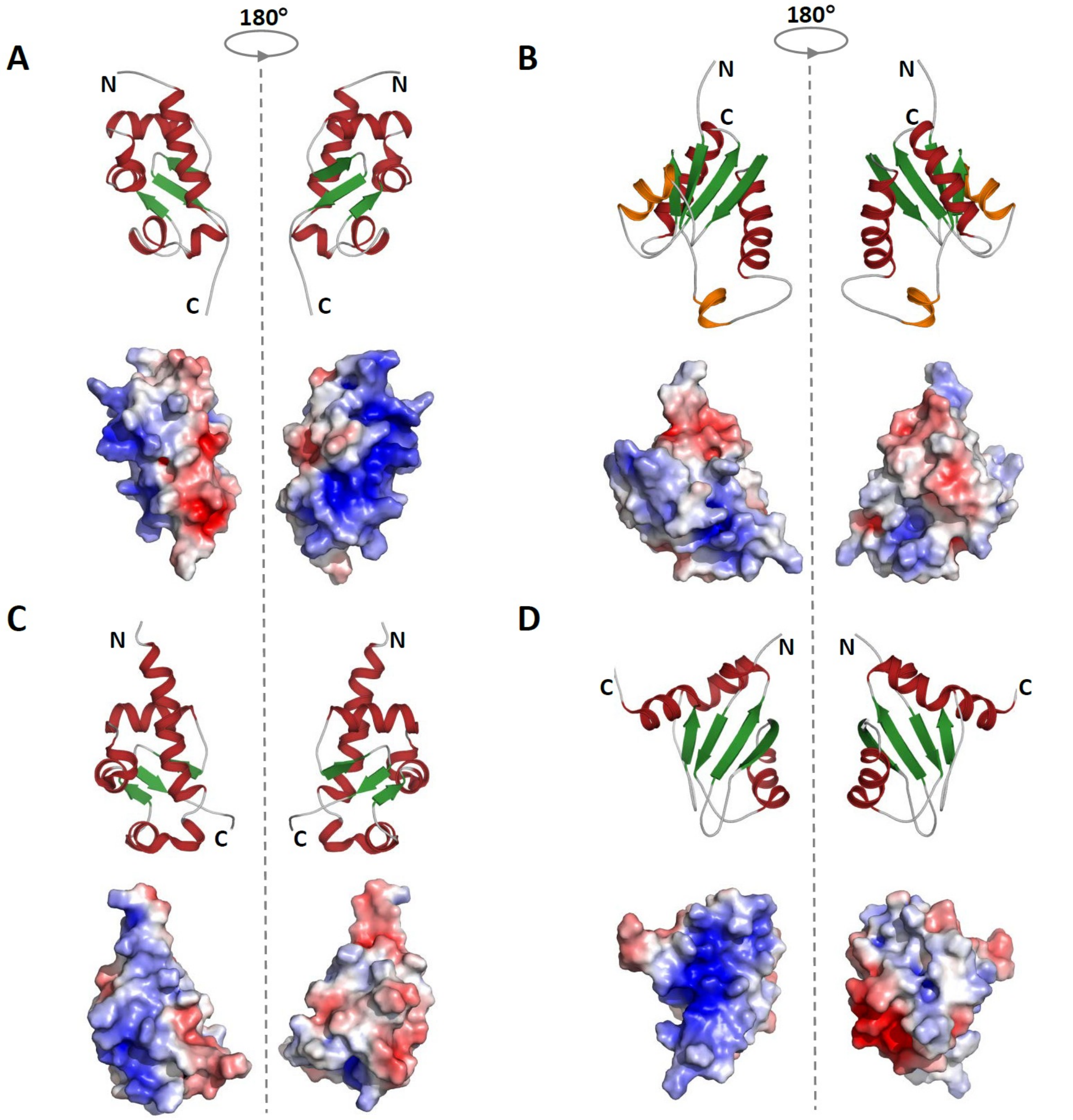

Figure S6. Structural analysis of HsLARP6 LaM and RRM1. Electrostatic surface potential of HsLARP6 (A) LaM and (B) RRM1 in comparison with HsLa (C) LaM and (D) RRM1. The homologous domains are shown in the same orientation. The orientations of the left-hand column for each domain are similar to those in Figure 2. The electrostatic surface potentials are red and blue for acidic and basic regions respectively and were generated with PyMOL implementing the APBS tool with standard parameters for the dielectric constants and using a linear Poisson-

Boltzmann equation. Interestingly, the face of the LaM opposite from the hydrophobic pocket in HsLARP6 is significantly more positively charged than in HsLa.

Figure S7. Analysis of the interaction of HsLARP6 with 48nt RNA. (A-O) ITC experiments showing the normalised binding curves of the following interactions: (A) 32nt RNA with HsLARP6 La module; (B-O) 48nt RNA with a range of mutants: (B) W85A, (C) K86A, (D) Q99A, (E) F102A, (F) Y103A, (G) D112A, (H) F114A, (I) F135A, (J) N180A, (K) L187A, (L) K196A, (M) I260A and (N) E262A; (O) 48nt with the longer HsLARP6 La module HsLARP6(74-313).

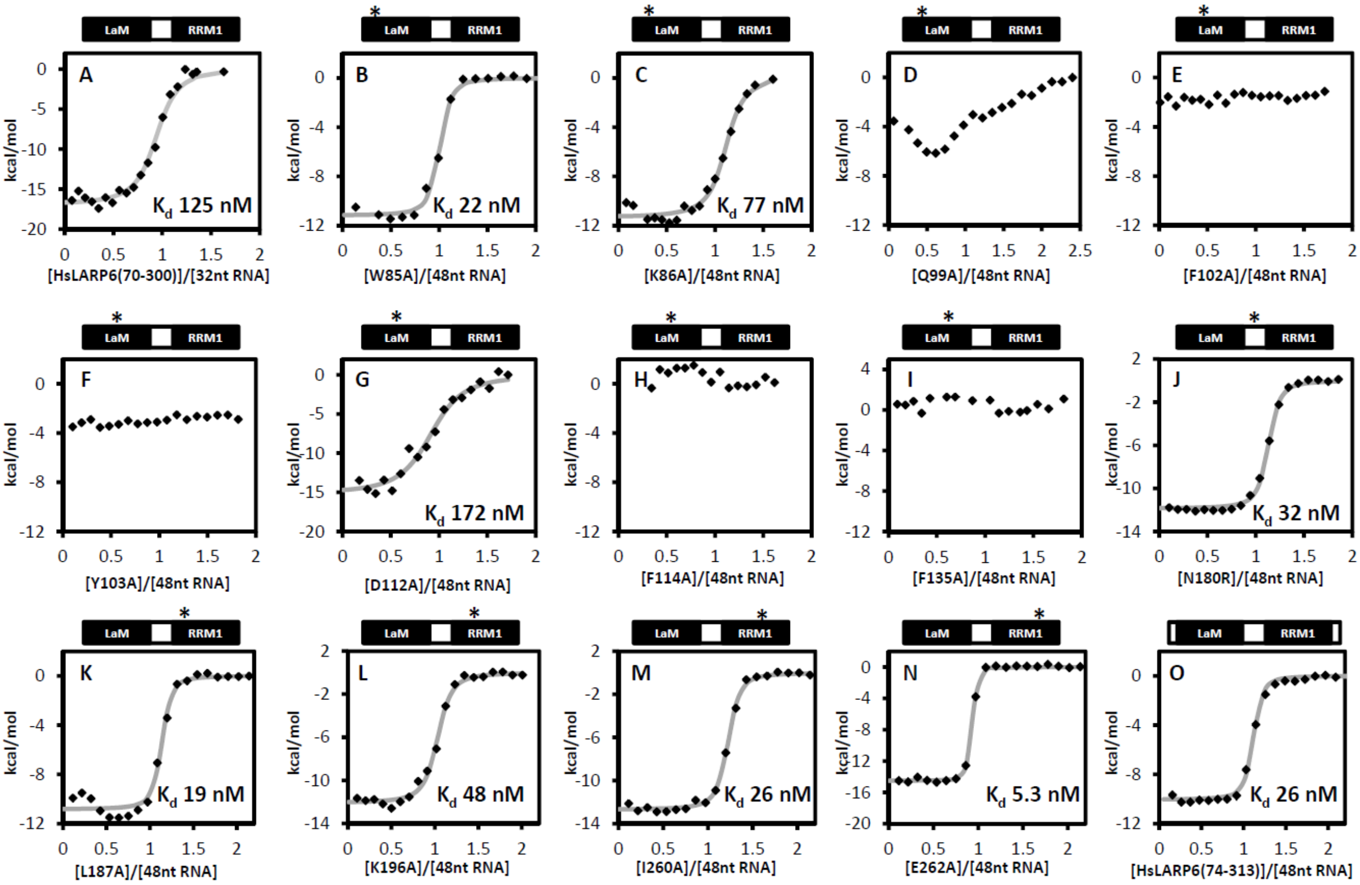

Figure S8

A

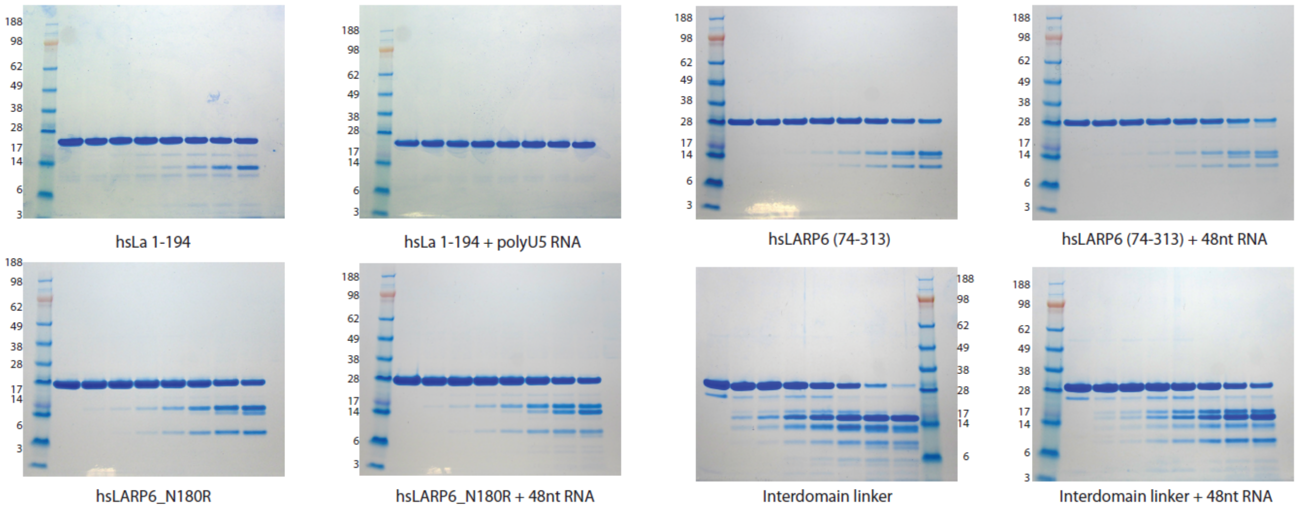

B

Protein	Fragment Mw (Da)	Fragment Boundaries	Cleavage Site
**HsLa(1-194)**	12564.6<br>12834.5	1-109<br>1-111	K109<br>R111
**HsLARP6N180R**	13688.6<br>16134.5<br>10708.3	70-184<br>70-205<br>206-300	K184<br>K205<br>K205
**HsLARP6(74-313)**	15776.4<br>13329.7<br>12130.9	74-205<br>74-184<br>206-313	K205<br>K184<br>K205
**Interlinker chimera**	14071.1<br>14341<br>13693.7<br>13340.6<br>10707.8	HsLARP6 70-HsLa linker 109<br>HsLARP6 70-HsLa linker 111<br>180-300<br>180-297<br>206-300	HsLa linker K109<br>HsLa linker R111<br>HsLa linker R111<br>HsLa linker R111, HsLARP6 K297<br>HsLARP6 K205

Figure S8. Limited proteolysis analysis of the La modules of HsLARP6 and HsLa. (A) Limited tryptic proteolysis of HsLa La module (residues 1-194) and HsLARP6 (74-313), HsLARP6N180R and Interdomain linker chimera in the presence and absence of target RNA. HsLa La module is protected from proteolytic cleavage (occurring in the interdomain linker) in the presence of RNA, probably owing to the rigidification of the linker/module upon RNA interaction. HsLARP6 La module and N180R mutant on the contrary did not undergo changes in proteolytic cleavage upon RNA addition. (B) Mass spectrometry analysis of the proteolytic fragments cleaved in the absence of RNA. Of the fragments identified, HsLa La module cleaves solely within the interdomain linker. The HsLARP6 constructs all cleave at K205 within the RRM1 loop 1. HsLARP6(74-313) and N180R additionally cleave at K184 at the beginning of the

RRM1. In addition to K205, the HsLARP6-HsLa Interlinker chimera cuts also within the hsLa linker and at K297 at the end of RRM1, but a cleavage site at K184 was not observed.

Figure S9: Chemical shift analysis of HsLARP6 La module. The [^{1}H,^{15}N]-HSQC spectra of the isolated LaM, HsLARP6(70-183), and RRM1, HsLARP6(180-295) were superposed to the spectrum of the tandem La module domain construct, HsLARP6(70-300), in A and B respectively. The colour codes are indicated. The resonances that appear to experience chemical shift variation are circled and labelled. The analysis was conduced as described in the methods and the ^{15}N and $^{1}H_N$ chemical shift variations are reported in the table below.

Analysis of ^{15}N and $^{1}H_N$ chemical shift variations of isolated LaM and RRM1 compared with the La module							
Domain	Chemical shift variations		Unperturbed residues	Unclear			
	$0.05<\Delta\delta_{AV} \leq 0.1$	Resonances that move or disappear – for which $\Delta\delta_{AV}$ is impossible to quantify		Not determined because of spectral overlap	Resonances whose chemical shift vary slightly with protein concentration	Unassigned residues	Proline residues
**LaM**	E90, K111, W145, L152	H118, E162, N180	G73, G74, E75, N76, E79, D80, L81, E82, W85, K86, L92, L96, V97, D98, I100, E101, D106, E107, N108, L109, E110, D112, A113, L116, G125, V127, S128, V129, F135, K137, H140, D144, H150, A151, S154, S155, V156, L157, E159, L160, N161, D163, H164, R165, V167, R168, R169, T170, T171, V173	E77, R78, E84, D89, E91, I93, K94, K95, Q99, F102, Y103, F104, S105, F114, L115, K117, V119, L124, L132, T133, S134, V138, K139, L141, R143, R146, T147, T148, A149, K153, L158, K166, L175, E179	L131, T142, F176, N178	T70, A71, S72, Q83, R120, R121, N122, K123, Y126, K130, K136	P87, P88, P172, P174, P177
**RRM1**	S184, M185, L187, F221, R231, V266, E267, I291	L218, S228, K234, E238, D242, G254, I260, V261, E262, L290, K294	L181, Y189, D190, L191, Y192, A201, T202, T223, G225, S229, G236, L239, Q252, T255, E257, E258, A259, A268, A269, A272, T278, E279, S280, Q281, G282, G292, M293	S183, L193, S194, K196, L197, W198, A199, L200, R208, V209, Q210, E211, K212, V213, M214, E215, H216, L217, K219, L220, F224, V226, V230, I232, R237, I243, R244, R245, I246, L247, S248, R249, Y250, V253, Q256, E264, I270, K271, H273, E274, F275, M276, I277, K283, E284, Q285, M286, K287, A288	L186, V188, I227, L233, F263, E 265, V289, G222	Q204, K205, N206, G207, S251	P182, P195, P203, P235, P240, P241, P295

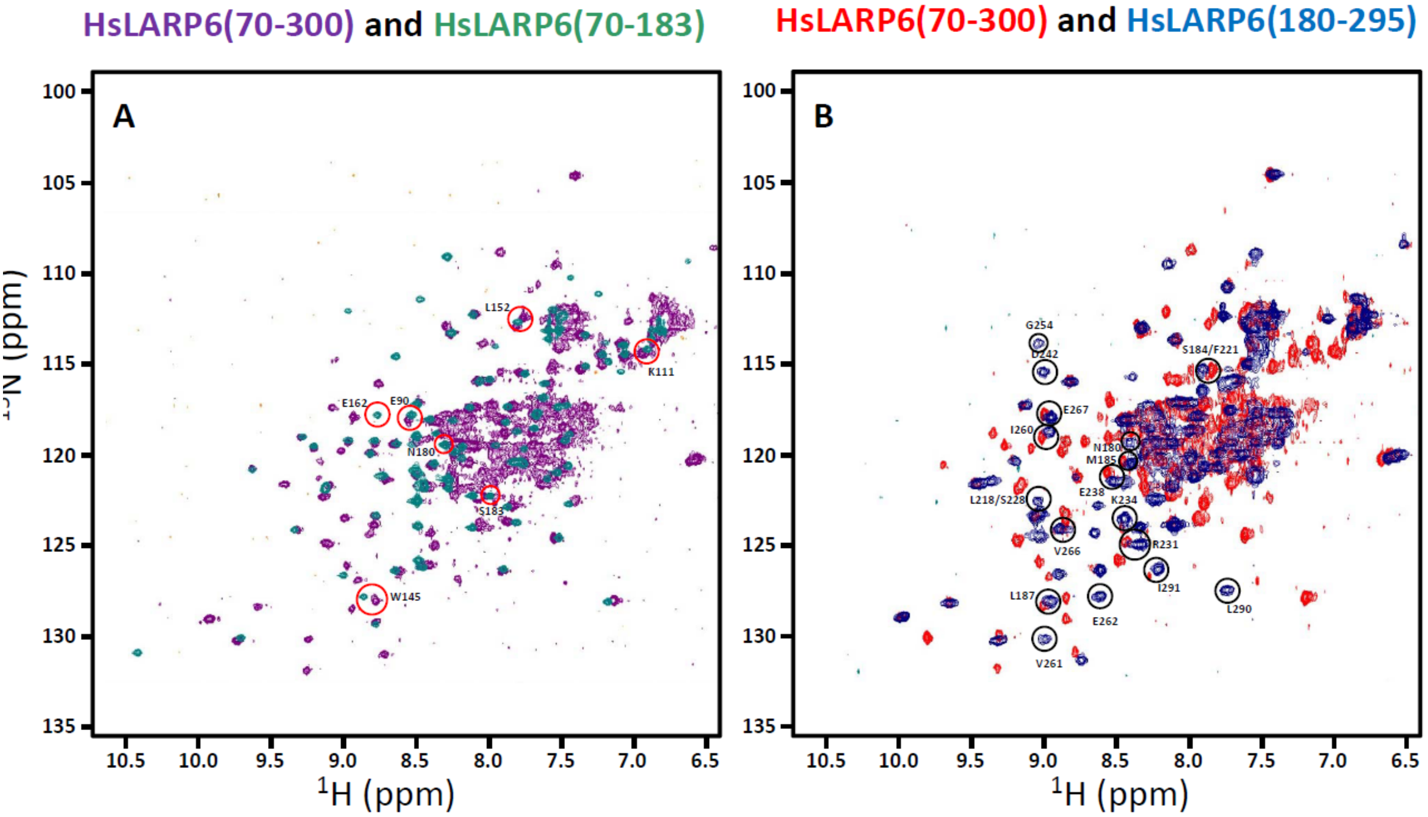

Buffer: 20 mM Tris pH 7.25, 100 mM KCl,
50 mM L-Arg/L-Glu, 1 mM DTT

Buffer: 20 mM Tris pH 7.25, 100 mM KCl, 1 mM DTT

Table S1. Thermodynamic parameters of the association of HsLARP6 with 48nt RNA and 32nt RNA in varying KCl and $MgCl_2$ concentrations at 25°C.

Interaction	n	Kd (nM)	ΔH (kcal)	-TΔS (kcal)	ΔG (kcal)
HsLARP6(70-300)/32nt RNA in 0 mM MgCl2	0.8	167	-13	3.8	-9.2
HsLARP6(70-300)/32nt RNA in 2 mM MgCl2	1.0	142	-14	4.7	-9.3
HsLARP6(70-300)/32nt RNA in 5 mM MgCl2	1.0	125	-17	7.6	-9.4
HsLARP6(79-300)/32nt RNA in 10 mM MgCl2	1.0	111	-16	6.5	-9.5
HsLARP6(70-300)/32nt RNA in 120 mM KCl	0.8	143	-14	4.7	-9.3
HsLARP6(70-300)/32nt RNA in 200 mM KCl	0.9	137	-11	1.7	-9.3
HsLARP6(70-300)/48nt RNA in 0 mM MgCl2	0.8	60	-15	5.2	-9.8
HsLARP6(70-300)/48nt RNA in 2 mM MgCl2	0.8	57	-12	2.2	-9.8
HsLARP6(70-300)/48nt RNA in 5 mM MgCl2	0.8	48	-11	1.0	-10.0
HsLARP6(70-300)/48nt RNA in 10 mM MgCl2	0.8	50	-12	2.1	-9.9
HsLARP6(70-300)/48nt RNA in 120 mM KCl	1.0	50	-13	3.1	-9.9
HsLARP6(70-300)/48nt RNA in 200 mM KCl	1.0	50	-13	3.1	-9.9

The errors on the reported K_d and ΔH are between 5-15%
